# Supplementary material for: Active mobility and mental health: A scoping review towards a healthier world
Source: Glob Ment Health (Camb). 2023 Nov 21;11:e1. doi: 10.1017/gmh.2023.74 (PMC10882204; doi:10.1017/gmh.2023.74)
Supplement: Scrivano et al. supplementary material 2 — Scrivano et al. supplementary material [file S2054425123000742sup002.docx]

The sheet “Lit_Rev” collect all the extracted data. The other sheets contain categorised information (e.g., study characteristics; sample; mental health output, etc.)

**Author(s)**

If there is only one author, reporting their name fully.

If there are two authors, reporting their names fully.

If there are more than two authors, reporting only the first name and “et al.,”.

**Year**

Reporting year of publication.

**Study Design**

Reporting the type of study design as described by the authors.

**Study Duration**

For cross-sectional designs, reporting the data collection period if it is equal to or longer than one year.

For longitudinal designs, reporting the baseline and follow-up year date.

For experimental designs, reporting the data collection year date(s).

**Methodology**

For cross-sectional and longitudinal designs, reporting the methodological approach if specified by the author.

For experimental designs, reporting the baseline and follow-up measurements.

**Intervention**

Only for experimental designs; reporting the intervention undertaken by the Intervention Group as described by the authors.

**Original Study/Project**

Reporting the original study or research project of which the paper is part, as stated by the authors.

“Stand-alone” codes for a study implemented independently, not as part of a larger project.

**Country – City/Region**

Reporting the location where the sample was recruited, the study conducted, and the intervention implemented as stated by the authors.

**Sample size**

Reporting the number of participants. “ND” codes for missing values.

**Inclusion age range**

Two columns reporting age max and min.

“199” codes for missing maximum age values.

“ND” codes for missing age values.

“*n* age” was used to code *mean* age if the study did not report the full age range.

**% women**

Reporting percentage of women in the sample.

“ND” codes for missing sex/gender values.

**Participants characteristic**

The research question of this review included only non-clinical adult samples. As a result, all the participants were considered “healthy adults” in each study. Thus, it is not specified in the table.

Reporting info about particular population cases if specified by the related study (e.g., university employees, community-dwelling older adults, etc.).

“working adults” codes for a sample of adults with a job.

“commuters to work” refers explicitly to people that travel to their workplace as a destination.

**MH Output (Mental Health Output)**

Reporting mental health outcomes measured in each study. Each was coded according to the methodology specified in section “3. Materials & Methods” of the present review as categorised in the present scoping review (e.g., physical self-efficacy is coded as “self-efficacy”; health-related quality of life is coded as “quality of life”).

**MH Measures (Mental Health Measures)**

Validated instruments are coded through the acronym (specifics about the measurements in APPENDIX A).

1-item questions are fully reported.

The sign “+” indicates that the outcome was measured with two or more tools, but the final score refers to one overall result.

**Mobility mode(s)**

Reporting what means of transportation are investigated by each study.

“Multimodes” codes for studies examining different mobility modes (e.g., car, public transport – metro, tram, bus, train – motorbike, cycling and walking).

“walking and cycling” codes for studies examining only Active Mobility/Travel/Commuting/transport.

If the study investigates one specific means of active transport, the mode is coded as “walking” or “cycling” accordingly.

Only one study categorised “active travel” as cycling or public transport and walking as a multimodal trip and it was coded specifically as “cycling and public transport (including walking)”.

**Purpose of AM (Purpose of Active Mobility)**

“Transportation” codes for active travel trips with general transport purposes (to and from work, study, chores, caring duties, shops, meeting or visiting family and friends).

“work/education” codes for travel to the workplace or educational sites, such as the university.

**AM Measure (Active Mobility Measures)**

Validated instruments are coded through the acronym (e.g., “IPAQ”; specifics about the measurements in APPENDIX A).

1-item questions are fully reported (e.g., “Have you ever used PBSP?”).

The sign “+” indicates that the outcome was measured with two or more tools, but the final score refers to one overall result.

“Frequency” codes for how often in days. When the study reports it, we specify the time range in the attached comments (e.g., “in a week” or “in the last month”).

“Duration” codes for the time in minutes per each trip.

“Distance” codes for the length covered during the trip.

Every study examined travel mode (exposure). To avoid overfilling the table with obvious info, “Mode” was used only for studies investigating the participants’ mode without asking about other trip characteristics.

Extra info was included in the attached comments (e.g., telephone or web-based survey or a diary) and Appendix A file.

**Main Results**

Reporting the main findings about active mobility and the specific mental health outcome per each study, summarising in a few words.

If explicitly reported, moderators are recorded in brackets (e.g., symptoms levels worsened if over 15 minutes of active travelling).

**AM benefits MH (Active Mobility benefits Mental Health)**

“No” codes for a non-significant relationship between active mobility and the specific mental health outcome.

“Yes+” codes for a significant positive relationship between active mobility and the specific mental health outcome (the two factors increase or decrease together).

“Yes-” codes for a significant negative relationship between active mobility and the specific mental health outcome (when one increases, the other decreases).

Particular cases are highlighted as follows:

W= the results are significant only for walking, or the study only investigates walking;

C= the result is significant only for cycling, or the study only investigates cycling;

M= the result is significant only for men.

**Analysis type sig. test**

               What test if any.

               For longitudinal studies without a control group, consider modelled regression where present.

               For each study, before detailed inspection of the contents, the following search strings were used to determine the type(s) of analysis present: effect size, t test, ANOVA, regression, correlation.

**N outcomes**

Number of distinct outcomes relating to mental health, reported quantitatively, whether in tables or text. If both summary and subscale scores are presented, show the results as N. summary (N. subscale).

**N models**

               Number of parallel regression models reported per reported outcome.

**N partitions**

               Number of subpopulations reported, e.g. (all participants, men, women) counts as 3.

**p-value**

               What was the quoted p-value for the outcome, irrespective of its method of calculation. If only the threshold value is quoted (e.g. p<0.05) use threshold value. If value quoted as 0.000 or similar use largest value compatible with number of significant figures shown and presumed rounding. See <https://doi.org/10.1136/bmj.d2304> for conversion of mean/CI to p-values

**N for p-value**

               What was the sample size used for calculating the p-value. If possible, quote numbers for each sub-groups (e.g. two value for a t-test). If this value is not recorded for the analysis of interest, use the value (or values) quoted for the study as a whole.

**Sign**

Code direction of effect so that active mobility is better independent of the outcome, e.g. +1 for stress means that those participating in active mobility are less stressed than passive travellers, while +1 for affect means greater positive affect or reduced negative affect. Sign should also be included for non-significant results.

**Raw data**

               What data is present (if different from mental health output), e.g. subscales with number appearing

**Raw data type**

               Form of raw data

**Largest covariate**

               If calculable, the largest reported covariate or confounding variable. If result on whole study population is present, only consider this. If only subgroup analysis present, which outcome and subgroup. If multiple subgroups e.g. sex and timepoint, work first by time (whole group, m.s. time point), then other factors. Only pick largest, and stop at first level producing result. Choose the most significant variable even if this is not the largest coefficient, unless there are reasons for discounting this.

**Largest PF outcome**

               If calculable, otherwise all with equivalent sig. level. If result on whole study population is present, only consider this. If only subgroup analysis present, which outcome and subgroup. If multiple subgroups e.g. sex and timepoint, only pick largest.

**Effect size**

               If effect size is calculated, what is it? Check tables, and text search for the string “effect size”. Do not impute effect sizes.

**Form of ES (Form of Effect Size)**

               What type of calculated effect size is present
