# Supplementary material for: Active mobility and mental health: A scoping review towards a healthier world
Source: Glob Ment Health (Camb). 2023 Nov 21;11:e1. doi: 10.1017/gmh.2023.74 (PMC10882204; doi:10.1017/gmh.2023.74)
Supplement: Scrivano et al. supplementary material 4 — Scrivano et al. supplementary material [file S2054425123000742sup004.docx]

APPENDIX A – Active transport modes and Mental Health outcomes

Each study is categorised per outcome and design. Per each study, this file will describe the authors’ name and year, the country, the original study, the exposure factor and outcomes with related measures, intervention details in case of experimental design, and the final results about the relationship between the variables in the exam. We also cared to explain when there might have been confusion between the name of exposure/outcome and the measure used. Some info may be repeated since each study might encompass more than one outcome, as listed in Table 1; however, we aimed for a detailed and comprehensive information set.

1. Mental Health and Mental Well-Being

CROSS-SECTIONAL:

In 2007, Ohta and colleagues (Ohta *et al.* 2007) conducted a survey questionnaire as part of a worksite mental health promotion in Kyushu (Japan). The study, published in 2007, investigated the association between walking or cycling to work (Do you walk or cycle to work? + Duration) and people’s mental health, measured through the General Health Questionnaire-28 (GHQ; Goldberg, 1978). Furthermore, the results demonstrate that commuting on foot or by bike significantly benefits mental health, but this was significant only in men.

In 2011, Hansson and colleagues (Hansson *et al.* 2011) evaluated the relationship between commuting mode (How do you usually get to work?”), time (“How long does it take to get to work?”), and health outcomes. These were perceived sleep quality, everyday stress, exhaustion, mental health, self-rated health, and sickness absence. The General Health Questionnaire -12 (GHQ; Goldberg et al., 1997) was used to evaluate mental health. Mental health was not significantly associated with commuting. However, the authors highlight the “healthy commuter effect” that explains the bias where car commuters might face poor health conditions or more stressful situations; this could mean that unhealthy people are less likely to start or continue to commute actively.

In 2013, Humphreys and colleagues (Humphreys *et al.* 2013) examined the relationship between active commuting (frequency+duration) and mental well-being. Although the outcome is called mental “well-being”, the measure is still the Mental Component Score (MCS) from the SF-8 (Ware *et al.* 2001). Surprisingly, this study showed no relationship between these variables. Data came from the Commuting and Health in Cambridge study (Ogilvie *et al.* 2010).

In 2016, Chng and colleagues (Chng *et al.* 2016) used data from wave two of Understanding Society - UK Household Longitudinal Study (2010/2011; previously known as British Household Panel Survey BPHS) to examine the relationship between what they called subjective well-being and commute mode (“How do you usually get to your place of work?”). Subjective well-being was assessed by measuring life satisfaction and “mental distress” with the General Health Questionnaire -12 (GHQ; Goldberg et al., 1997). However, no commute mode was associated with significant changes in GHQ scores.

The same year, Mason and colleagues (Mason *et al.* 2016) examined the relationship between physical activity domains (household, occupational, active travel, leisure and sport, and family activities) and mental health and well-being. The study was conducted in Glasgow, Scotland, as part of the GoWell Research and Learning Programme (GoWell 2010). It is the only study separating mental health, measured with the Mental Component Score (MCS) from SF-12 ( Ware et al., 2002), and positive mental well-being, measured with the Warwick-Edinburgh Mental Well-being Scale (WEMWBS; (Tennant *et al.* 2007). The survey asked participants to “Rank 5 PA domains in order of how much you do each of them’”. The results recorded higher levels of both mental outcomes significantly related to active travel physical activity.

In 2020, Clark and colleagues (Clark *et al.* 2020) used data from six waves of the Understanding Society - UK Household Longitudinal Study (2009/10 – 2014/15; University of Essex, 2016) (previously known as British Household Panel Survey BPHS) to analyse the impact of commuting in terms of duration and mode on what they called subjective well-being, measuring self-reported health, exhaustion and mental health (GHQ-12; Goldberg et al., 1997). The results reported a non-significant association between mental health scores and active travel modes. The only significant result in commuting mode was that people travelling by bus reported poorer mental health than car drivers for every additional commuting minute.

LONGITUDINAL:

In 2006, Lampinen and colleagues (Lampinen *et al.* 2006) examined the role of *mobility status* as a predictor of mental well-being. The original project was an eight-year follow-up study called the Evergreen project among older adults (aged 65-84) in Jyvaskyla, Finland (Heikkinen 1998). Mobility status was defined as “the capability to climb stairs or walk two kilometres non-stop” (Guralnik *et al.* 1993). The summary of the following factors gave the mental well-being score:

- *Depressive symptoms:* The Revised Beck’s Depression Inquiry (RBDI, Raitasalo, 1995)
- *Anxiety*: from fairly relaxed to continually anxious and distressed; question part of the RBDI
- *Loneliness*: ‘Do you think you are lonely?’
- *Self-rated mental vigour*: ‘How would you describe your self-rated mental vigour at the moment?’
- *Meaning in life*: ‘Right now, how meaningful do you consider your life?’

At baseline (1988), better mobility was associated with better mental well-being. At follow-up (1996), both baseline mental well-being and better mobility status predicted higher scores in mental well-being.

In 2014, Martin, Goryakin and Suhrcke (Martin *et al.* 2014) conducted a study to counterprove Humphreys’ results (Humphreys et al., 2013). Using data from the British Household Panel Survey (BHPS, 18 waves from 1991/2 to 2008/9; (Taylor *et al.* 2001), the authors explored the relationship between active travel (“What usually is your means of travel to work?’) and *psychological well-being*. Despite the name, the instrument used to measure the outcome was the General Health Questionnaire-12 (GHQ; Goldberg et al., 1997). After adjusting for confounding factors like work, residence and health status, they found a significant association between GHQ-12 scores and active travel and public transport compared to car travel. Lastly, switching from car to active travel was related to improved well-being. It was also associated with a reduced likelihood of experiencing two specific psychological symptoms than car drivers (as the GHQ-12 reports, “being constantly under strain or unable to concentrate”).

In 2016, Mytton and colleagues (Mytton *et al.* 2016) conducted a longitudinal study built on Humphreys’s cross-sectional evidence (2013). The authors analysed data from the Commuting and Health in Cambridge study (Ogilvie et al., 2010) to explore longitudinal associations of maintenance and changes in active commuting, physical and mental well-being, and sickness absence. Weekly time spent walking or cycling to work was recorded on a 7-day travel diary in terms of frequency (number of trips) and duration of each trip. Maintenance of walking or cycling to work was assessed as ‘weekly walking/cycling time>0min at both baseline and follow-up. Mental well-being was measured with the Mental Component Score-8 (MCS from SF-8, Ware et al., 2001). Commuters maintaining cycling as travel behaviour reported better mental well-being at follow-up - no significant associations between any change in cycling and MCS. Also, no significant associations were observed for walking.

In 2018, the authors working on the Physical Activity through Sustainable Transport Approaches project (PASTA project, (Gerike *et al.* 2016) analysed the data collected between 2014 and 2016 to evaluate the association between transport modes (“How often do you currently use each of the following methods of travel to get to and from places?”) and health and social contact measures in seven European cities (Avila-Palencia *et al.* 2018). The authors evaluated the impact of single and multiple transport modes on the outcomes. Perceived stress, vitality and mental health were components of overall mental health outcomes. The Mental Health Inventory (MHI; the 5-item mental health scale of SF-36; (Ware *et al.* 1993) scores reported that cycling was associated with higher mental health than other transport modes in unimodal and multimodal trips.

In 2020, Kroesen and De Vos (Kroesen and De Vos 2020) examined the “health commuter effect” mentioned above (Hansson et al., 2011). The authors used data from 10 waves (2007-2017) of the Longitudinal Internet studies for the Social Sciences (LISS; www.lissdata.nl) panel, representing the Dutch population. They measured the effect of active travel on mental health and vice versa. First, active travelling was assessed with one question “If you look back on the past 7 days, how many of those days did you spend at least 10’ walking?”. In addition, mental health was measured with the Mental Health Inventory-5 (MHI-5; Berwick et al., 1991). The analysis discovered that active travel significantly impacts mental health, while the opposite direction was not statistically significant. However, this study only asked about active travelling as walking without specifying between leisure and utilitarian purposes.

QUASI-EXPERIMENTAL:

In 2017, Page and Nilsson (Page and Nilsson 2017) conducted a quasi-experimental study implementing a behaviour-changing intervention in a UK-based workplace. Participants were invited to change their commute behaviour to work using electrically assisted bikes (e-bikes). It was a quasi-experimental design since the authors allowed participants to borrow and use an e-bike for up to 5 months, as and when they pleased. Both qualitative and quantitative data were collected. Specifically, a weekly diary was used to record the commute to the workplace, noting the frequency and duration of the active trips. Also, the study used the General Health Questionnaire-12 (GHQ; Goldberg et al., 1997) as “an indicator of physical health” (even if it is a mental health/illness instrument). At the end of the intervention, the active transport group reported significantly higher GHQ-12 scores than the passive travel group.

In 2021, Jacob and colleagues (Jacob *et al.* 2021) analysed data from seven Understanding Society - UK Household Longitudinal Study waves from 2009 to 2016. Asking about the travel mode (‘How do you usually get to your place of work?’), the large sample was stratified into ‘treated’ for those who changed commute mode and ‘controls’ for those who did not. Specifically, SF-12 (Ware et al., 1996) was the health measure summarising mental (MCS - Mental Component Score) and physical scores. The main results indicate a significant increase in physical and mental health for commuters who switch from car to active travel, especially women. Conversely, a decline in reported mental health is registered for those who changed from active to public transport. Notable impacts on health or satisfaction did not accompany car and public transport changes.

2. Quality of Life

CROSS-SECTIONAL:

In 2009, Cerin and colleagues (Cerin *et al.* 2009) measured the relationship between four physical activity domains (leisure, household, occupational, and transport) using the IPAQ (Craig *et al.* 2003) and mental well-being. Despite a different terminology, the outcome was measured using the SF-12 (Ware et al., 1996). Data came from the Physical Activity in Localities and Community Environments (PLACE) study, Adelaide, Australia. Transport physical activity was unrelated to mental well-being in all demographic groups. However, all physical activity domains showed significant group differences according to age and gender.

In 2010, Jurakić and colleagues (Jurakić *et al.* 2010) performed one of the first studies about the relationship between different physical activity domains (job-related, domestic, transportation, leisure-time) using the IPAQ (Craig et al., 2003) and health-related quality of life (HRQoL) in a sample of Croatian inhabitants. HRQoL was assessed using the SF-36 (Ware and Gandek 1998). After adjusting for confounding factors related to physical activity and HRQoL (size of settlement, age, educational level, cigarette and alcohol consumption, BMI) (Trost *et al.* 2002; Vuillemin *et al.* 2005), the authors described an inverse relationship between transportation physical activity and HRQoL. Moreover, they suggested that future research should assess domain-specific PA effects on this outcome.

In 2012, Pucci and colleagues (Pucci *et al.* 2012) conducted a similar analysis in a sample from Brazil. They evaluated the relationship between leisure-time or transport physical activity (active commuting) with quality of life domains (physical, social relations, environmental and psychological). Active commuting was only related to walking and measured using the IPAQ (Craig et al., 2003). Quality of life was assessed using the short form of the World Health Organisation Quality of Life questionnaire in Portuguese (WHOQoL-BREF; 26 items; Fleck et al., 2000; The Whoqol Group, 1998). The results showed that transport physical activity had a linear relationship with the physical domain of quality of life and only in men.

In 2013, Gómez and colleagues (Gómez *et al.* 2013) narrowed previous results by studying the relationship between Health-Related Quality of Life (HRQoL) and two physical activity domains (leisure-time and transport) in women residents in Colombia. HRQoL was assessed using the SF-8 (QualityMetrics 2008), and transport physical activity was related to walking for transportation (WT; IPAQ - Craig et al., 2003). WT and both mental and physical dimensions of HRQoL showed a negative relationship, so the more women used walking to transport, the less they rated their quality of life.

In 2019, Scarabottolo and colleagues (Scarabottolo *et al.* 2019) implemented a cross-sectional study to investigate the relationship between physical activity domains (work/occupational, leisure-time and transport, sports/gym) and HRQoL in older adults in Brazil. Interestingly, transportation physical activity was considered within the same domain as leisure-time physical activity (four items to examine “leisure-time, corresponding to activities in the off-work time and regarding active commuting by cycling or walking”; Baecke et al., 1982). This probably happened because of the sample’s age (over 60 years old) and their life habits. HRQoL was measured using the SF-36 (Ware et al., 1993). Overall, higher levels of physical activity were associated with better HRQoL. In particular, no significant association was found between being highly active in the leisure-time domain or during active travel and HRQoL.

In 2022, Cobbold and colleagues (Cobbold *et al.* 2022) broadened these results. They examined the impact of multimodal trips on people’s well-being in Sydney, Australia. Data were collected from the Sydney Travel and Health Study (STAHS; Rissel et al., 2013). The travel behaviour was recorded online through a 7-day diary, and a smartphone application was available for the participants; however, its usage was optional. They asked people to record activity type, origin, destination, departure/arrival time, mode, and, importantly, if the trips were multimodal, recording each leg of active travel taken. They specified that quality of life was considered an indicator of “individual travellers’ general health and wellbeing” to sum up previously studied outcomes (such as travel satisfaction and subjective well-being). Quality of life was assessed using the short version of the World Health Organisation Quality of Life questionnaire validated in an Australian sample (WHOQoL-BREF; Murphy et al., 2000; The Whoqol Group, 1998). The most common transport mode was walking, followed by private motor vehicles. All the public transport and nearly half of the walking trips were multimodal. Bicycle trips were unimodal, virtually always. The sample reported better quality of life scores when taking unimodal active travel trips. The same result was recorded for multimodal public transport and either walking or cycling trips, compared to unimodal private motor trips.

LONGITUDINAL:

In 2022, Scarabottolo and colleagues (Scarabottolo *et al.* 2022) replicated the cross-sectional study from 2019 over two years. Overall, the sample increased their domain. It was the first and only study we found where ‘locomotion’ is used for active travel. In particular, increased leisure/locomotion physical activity was associated with lower functional capacity scores and improved vitality and mental health, specific domains of HRQoL measured by the SF-36 (Ware et al., 1993). In conclusion, the authors suggested that practising leisure/locomotion physical activity might positively impact some aspects of quality of life, specifically mental health and vitality, and negatively influence others (decreased functional capacity).

CONTROLLED-TRIAL:

In 2008, de Geus and colleagues (De Geus *et al.* 2008) implemented the first non-randomised controlled study about the effect of cycling to work on mental health outcomes and quality of life. This study examined the influence of a 1-year lifestyle intervention (cycling to work) on HRQoL, other than the cardiovascular-heart disease (CHD) risk, in adults living in Flanders, Belgium. The sample was divided according to home-work distance (the control group lived closer than 2km). In addition, participants recorded their trip on a 7-day diary, specifying the bouts/week and the duration. The intervention group subjects were asked to cycle to work at least three times/week; the control group subjects were requested not to change their living habits. Other than at baseline, the study included two follow-up measurements at 6 and 12 months. This study also used CicloMaster, a German distance recorder. In addition, HRQoL was assessed using the SF-36 (Ware et al., 1993). Overall, the authors concluded that cycling to work would likely improve HRQoL and positively influence CHD risk factors.

RCT:

In 2000, Mutrie and colleagues (Mutrie *et al.* 2000), for the first time, presented an RCT wondering if active commuting could improve quality of life. They implemented a cognitive-behavioural intervention (pre-tested written and visual interactive materials) to increase active commuting behaviour and, secondarily, evaluate if this increase could impact the quality of life levels measured through the SF-36 (Ware et al., 1993). Participants received a baseline questionnaire measuring demographic variables, stage of behaviour change, and 7-day recall physical activity (SPAQ; Lowther et al., 1999). Follow-up questionnaires were administered after 3, 6, 9, and 12 months from baseline. Firstly, the intervention was successful, and the intervention group increased their active commuting levels. Also, after three months, those who had increased their active mobility levels tended to improve both physical and psychological aspects of their quality of life (vitality, emotional health, mental health, and body pain). The authors concluded that it was modest evidence of the impact on quality of life, but more sensitive measures were required to explore these dynamics better.

Two years later, Mutrie and colleagues (Mutrie *et al.* 2002) implemented a self-help intervention to promote active commuting and evaluated if this could be associated with overall health benefits. Once again, the study protocol was an RCT; the sample was adults from workplaces in Glasgow, Scotland. Using the same instrument pack implemented in the previous experiment, they noted that, after six months, the intervention group increased their walking levels almost twice the control group. Additionally, the intervention group significantly improved their scores in reported Mental health, Vitality and General Health subscales of SF-36 between baseline and six months. So, the authors concluded that walking as a means of transport could improve the overall quality of life. However, the intervention was not successful in increasing bicycling.

In 2008, Baker and colleagues (Baker *et al.* 2008) evaluated the impact of a 12-weeks consultation and pedometer-based walking intervention (“Walking for Well-being in the West, WWW study”; Fitzsimons et al., 2008) on step counts and health-related outcomes as part of the Scottish Physical Activity Research Collaboration (SPARColl). The tools used to measure walking were the pedometer *Omron HJ-109E Step-O-Meter* (Omron Healthcare UK Ltd) counting steps/day and the IPAQ (Craig et al., 2003). Participants also recorded the duration of their trips (walking time). Affect and quality of life were the health-related outcomes included. The latter was measured with the Euroqol EQ-5D instrument (The EuroQoL Group 1990). The intervention succeeded: control group subjects displayed no significant change in steps/day over time, while intervention group subjects had an increase that exceeded the expected/recommended goal. However, there was no significant effect on any quality of life score.

In 2020, as part of the GISMO (Geographical Information Support for healthy Mobility; Reich et al., 2020), Neumeier and colleagues (Neumeier *et al.* 2020) evaluated the impact of active commuting on HRQoL and absence days from work in Salzburg, Austria. HRQoL was defined as “how well a person functions in one’s life and his or her perceived well-being in physical, mental and social domains of health” (Hays and Reeve 2010). Web-based travel diary provided data for the distance, duration, and frequency of participants’ daily commuting trips. Additionally, they were asked to wear fitness watches, Polar M200 (also measuring location and heart rate; Loidl et al., 2020), for two weeks at the beginning and end of the intervention to validate data collected through the diaries. Sick-leave days were included in the study because they were considered a “major economic burden in many economies” (OECD 2010). The intervention group was asked to actively commute to work for 12 months, while the control group was asked not to change their habits. Also, the intervention group was divided into IG-C for those who cycled to work and IG-PT for those who used public transport (which also included walking), according to home-work distance, personal preference and available infrastructures. HRQoL was measured using the German version of the SF-36 (Bullinger 1995), and the sick-days number was provided by the employer referring to the year before the study. The intervention group showed significant positive changes in every subcomponent and total SF-36 scores compared to controls. Overall, active travel intervention showed a significant positive effect on the quality of life; specifically, IG-C had greater, more significant changes than IG-PT, although IG-C and IG-PT had no significant differences at baseline. Lastly, only IG-PT showed a significant decrease in sick-leave days.

3. Affect

CROSS-SECTIONAL:

In 2007, Gatersleben and Uzzell (Gatersleben and Uzzell 2007) measured the *affective appraisal* connected to mobility modes (measuring their usage frequency) as “sources of positive and negative affect when people use different travel modes”. *Affect* measure consisted of a 5-point Likert-type scale based on Russell and Lanius’s two-dimensional model of affect (Russell and Lanius 1984). The results showed that walking and cycling were considered relaxing and exciting modes (positive affects), while car commuters were the most stressed ones (negative affects).

In 2010, Rasciute and Downward (Rasciute and Downward 2010) used data from the Taking Part Survey (since 2005, DCMS, England). The survey collected data about 67 sports activities, recording participation in the last 12 months and the previous four weeks, the frequency and the duration of each activity. One of them was cycling for utilitarian purposes. ‘Happiness’ (positive affect) was employed as a synonym of *well-being*: “Taking all things together, how happy would you say you are?”. However, while walking had a statistically significant positive effect on positive affect, cycling for “utilitarian” purposes had a non-significant impact on happiness scores.

In 2014, the Office for National Statistics (Office for National Statistics 2014) examined the relationship between time and mode used to commute to work (“Do you work from home?”; “What is the travel time in minutes from home to work, one way?”) and ‘personal well-being’, defined as composed of happiness, anxiety, life satisfaction and worthiness. The results suggested that AT had a stronger relationship with the affective component (happiness, anxiety and life satisfaction) of *personal WB.* In addition, the survey asked, “Overall, how happy did you feel yesterday?”. In general, commuters to work were less happy than non-commuters. Moreover, those who used public transport (bus or train) had the lowest happiness levels; active travelling (walking and cycling) was associated with lower happiness levels than private vehicles, especially for commuting more than 15 minutes.

In 2015, Morris and Guerra (Morris and Guerra 2015) defined *affect* as a synonym of “mood”, both constructs related to “individual well-being”. This concept was measured by reporting happiness, sadness, tiredness, pain and stress levels on a scale of 0-6 (the paper does not note the items). The survey investigated only what travel mode participants used. The results showed that travel has little impact on the overall affect state, and the relationship with travel mode tends to be not statistically significant. However, cycling users are the happiest travellers.

In 2017, Lancèe and colleagues (Lancée *et al.* 2017) analysed commuters’ mood. The authors defined “mood” as the “affective component of total subjective well-being” or “the affective component of happiness”. Data were collected using the Dutch ‘Happiness Indicator’ (“GeluksWijzer”; Burger & Veenhoven, 2016). A Day Reconstruction Method (DRM; Kahneman et al., 2004) asked people to record “how happy they had felt during activities the previous day”. In addition, the survey asked what time of the day people commuted, using what mode, and with whom (or alone). Public transport was associated with the lowest happiness, while bike use with the highest. Hence, a significant positive relationship existed between active travel and positive affect.

In 2019, Kaplan and colleagues (Kaplan *et al.* 2019) implemented a study evaluating the relationship between bike use, positive mood, and positive self-concepts. The investigation measured bicycle use recurrence (Aarts *et al.* 1997) as an indicator of cycling travel habits. Self-concepts and mood were considered the eudaimonic and hedonic dimensions of cycling. Positive mood was measured with a scale adapted from the Profile Mood State Scale (Shacham 1983): positive mood states are associated with happiness and vigour, while negative ones are associated with tension and anxiety. The results highlighted a positive relationship between cycling, self-concepts and positive mood. Consequently, the authors concluded that cycling “generally contributes to *personal WB”.*

In 2019, Brainard and colleagues (Brainard *et al.* 2019) published the results of a cross-sectional analysis of data from the Adult Lives Survey 2016/2017 (Ipsos Mori 2018) of people aged 16-104 years living in England. Active travelling was investigated by asking how many days, in the last 28 days, they did “…any walking (or cycling) primarily to get from place to place (e.g., walking to work), rather than for health or recreation” of a minimum 10 minutes duration and the trip duration. Moreover, part of the sample received questions about “mood traits”. These questions investigated happiness (positive affect), anxiety, sense of worth (eudaimonia), life satisfaction, and personal effectiveness (self-efficacy) using the same items previously listed by the ONS study (Office for National Statistics 2014). The results showed that affect was not significantly related to active travel, either walking or cycling.

In 2021, Lira and Paez (Lira and Paez 2021) investigated *subjective well-being* (OECD 2013) in terms of how “people’s affective reactions to their experiences” changed regarding different means of transport in Santiago, Chile. Travel was assessed by asking, “What is your primary mode of travel for your regular commute?”. The reactions were assessed with the following values: *freedom, enjoyment, happiness, poverty, luxury, and status.* The item asked, “Please indicate the mode(s) of transport that you relate to the following feelings and concepts” for each value. The respondent would also name their regular mode(s) of transport. The authors wanted to examine the “consonance” (given by their affective reaction) between the ideal mode and the actual travel mode. Alignment between the two was a sign of better subjective well-being. At the same time, “dissonance” (Schwanen and Mokhtarian 2004) would be detrimental, especially for those who are “forced” to use active mobility modes (De Vos 2018). The results showed that public transport users had the highest dissonance, while active travellers had the lowest. Also, the former was mainly associated with *poverty*; the latter was associated with *freedom, enjoyment, and happiness.* The private motor vehicle was most associated with *Luxury and Status.*

LONGITUDINAL:

In 2019, Glasgow and colleagues (Glasgow *et al.* 2019) implemented an innovative study with a population of adults in the USA. Given the transient nature of mood, they wanted to overgo bias related to retrospective data and record mood states while the activity was ongoing. So, they used an experience sampling methodology (ESM) where participants were asked to record the details soon after the trip. The tools used were the smartphone app “Daynamica” to record the journeys (Mode+Frequency+Duration+Purpose+Activities) and the Travel Mood Scale (Glasgow *et al.* 2018). They explored the impact of activities during the journey, the purpose of the trip, and the environment types. Regarding travel mode, they found that active travel was associated with higher levels of positive mood than motorised vehicles.

QUASI-EXPERIMENTAL:

In 2017, Page and Nilsson (Page and Nilsson 2017) conducted a quasi-experimental study implementing a behaviour-changing intervention in a UK-based workplace. Participants were invited to change their commute behaviour to work using electrically assisted bikes (e-bikes). It was a quasi-experimental design since the authors allowed participants to borrow and use an e-bike for up to 5 months, as and when they pleased. Both qualitative and quantitative data were collected. Specifically, a weekly diary was used to record the commute to the workplace, noting the frequency and duration of the active trips. Also, participants were given a diary to record the commute’s impact on their affect (e.g., feeling tired and energized). Overall, the results indicated a positive change in reported affect from pre to mid-intervention among the active travel group, associated with changing from passive to active commuting mode.

RCT:

In 2008, Baker and colleagues (Baker *et al.* 2008) evaluated the impact of a 12-weeks consultation and pedometer-based walking intervention (“Walking for Well-being in the West, WWW study”) on step counts and health-related outcomes as part of the Scottish Physical Activity Research Collaboration (SPARColl). Affect and quality of life were the health-related outcomes included. The former was measured with the Positive and Negative Affect Schedule (PANAS; Watson et al., 1988), which is a self-report measure of positive (interested, alert) and negative (distressed, upset) feelings and emotions. The intervention succeeded: the control group displayed no significant change in steps/day over time, while the intervention group had an increase that exceeded the expected/recommended goal. However, the only significant result was a small to medium increase in positive affect reported by the intervention group.

4. Eudaimonia

CROSS-SECTIONAL:

In 2014, the Office for National Statistics (Office for National Statistics 2014) examined the relationship between time and mode used to commute to work (“Do you work from home?”; “What is the travel time in minutes from home to work, one way?”) and ‘personal well-being’, defined as composed of happiness, anxiety, life satisfaction and life worthiness, meaning a sense of purpose in life. The survey asked, “Overall, to what extent do you feel the things you do in your life are worthwhile?” Those who commute generally rated their daily activities less worthwhile than non-commuters. Moreover, public and active transport had lower scores than those who commuted using a private vehicle, especially for commuting longer than 15 minutes. After controlling for other individual characteristics, the analysis showed a clear negative association between commuting and personal well-being, although the results showed a stronger relationship with the hedonic component of *personal WB.*

In 2019, Kaplan and colleagues (Kaplan *et al.* 2019) implemented a study evaluating the relationship between bike use, positive mood, and positive self-concepts. The investigation measured bicycle use recurrence (Aarts *et al.* 1997) as an indicator of cycling travel habits. Self-concepts and mood were considered the eudaimonic and hedonic dimensions of cycling. The authors measured cycling self-concepts through statements based on four dimensions: physical, social, emotional (psychological) and contextual ability (self-efficacy) (Fleming and Courtney 1984). The results highlighted a positive relationship between cycling, self-concepts and positive mood. Consequently, the authors concluded that cycling “generally contributes to *personal WB”.*

In 2019, Brainard and colleagues (Brainard et al., 2019) published the results of a cross-sectional analysis of data from the Adult Lives Survey 2016/2017 (Ipsos Mori, 2018) of people aged 16-104 years living in England. Active travelling was investigated by asking how many days, in the last 28 days, they did “…any walking (or cycling) primarily to get from place to place (e.g. walking to work), rather than for health or recreation” f a minimum 10 minutes duration and the trip duration. Moreover, part of the sample received questions about “mood traits”. These questions investigated happiness (affect), anxiety, eudaimonia (sense of worth), life satisfaction, and self-efficacy (personal effectiveness) using the same items previously listed by the ONS paper (Office for National Statistics, 2014). The results showed that eudaimonia was not significantly related to active travel, either walking or cycling.

In 2021, Liu and colleagues (Liu *et al.* 2021) performed a similar investigation. They examined the commuting experience (travel behaviours in terms of daily commute mode choice) and aspects of hedonic (pleasure, happiness, satisfaction) and eudaimonic (a sense of purpose/meaning in life) well-being in Heze, China. *Eudaimonic WB* was assessed using the Flourishing Scale (Diener *et al.* 2010). The results indicated that hedonic well-being positively correlates with the journey experience and eudaimonia; however, eudaimonia and the journey experience were not directly related. The authors concluded that commuting experience is still indirectly related to eudaimonic well-being since both variables directly correlate with hedonic well-being.

QUASI-EXPERIMENTAL:

In 2017, Page and Nilsson (Page and Nilsson 2017) conducted a quasi-experimental study implementing a behaviour-changing intervention in a UK-based workplace. Participants were invited to change their commute behaviour to work using electrically assisted bikes (e-bikes). It was a quasi-experimental design since the authors allowed participants to borrow and use an e-bike for up to 5 months, as and when they pleased. Both qualitative and quantitative data were collected. Specifically, a weekly diary was used to record the commute to the workplace, noting the frequency and duration of the active trips. Also, the study measured *personal well-being* as a summary score of the Flourishing Scale (Diener *et al.* 2009), the General Health Questionnaire-12 (Goldberg et al., 1997) and personal affects recorded in a diary. The results showed a positive but non-significant association between active modes and flourishing scale scores.

5. Life Satisfaction

CROSS-SECTIONAL:

In 2014, the Office for National Statistics (Office for National Statistics, UK) examined the relationship between time and mode used to commute to work (“Do you work from home?”; “What is the travel time in minutes from home to work, one way?”) and ‘personal well-being’ as composed of happiness, anxiety, life satisfaction and worthiness. In addition, the survey asked, “Overall, how satisfied are you with your life nowadays?”. In general, commuters to work were less satisfied than non-commuters. Moreover, those who used public transport (bus or train) had the lowest life satisfaction levels; active travelling (walking and cycling) was associated with lower life satisfaction levels than private vehicles, especially for commuting more than 15 minutes.

In 2016, Chng and colleagues (Chng *et al.* 2016) used data from wave two of Understanding Society (UKHLS; 2010/2011). Commuting was investigated through one item (“How do you usually get to your place of work?”). Life satisfaction was measured using the global single-item question, “How dissatisfied or satisfied are you with your life overall?”. Although public transport was the most common travel mode, only walking was associated significantly with higher life satisfaction.

In 2017, Sener and Lee (Sener *et al.* 2017) analysed the El Paso Survey data. The survey investigated life satisfaction as the mental component of overall well-being. Terms like mental health, well-being, happiness and life satisfaction were used as synonyms and measured “how satisfied they are with their life”. The results reported a moderate positive association between active travel (measuring the frequency) and life satisfaction. However, life satisfaction levels decreased for older subjects. The authors hypothesized that this moderation might be due to the demanding physical effort required by active transportation, particularly cycling.

In 2018, McCarthy and Habib (McCarthy and Habib 2018) estimated the relationship between life satisfaction and travel behaviour using data from the Nova Scotia Travel Activity survey (NovaTRAC, Canada 2016). The survey contained a 24-hours activity diary (n° of trips, mode, distance, duration) and questions on health and attitudes towards transportation. The authors explained how life satisfaction could be interpreted as the cognitive aspect of subjective-wellbeing (also made of an affective component, named “happiness”). These authors measured life satisfaction by asking, “How would you describe your usual attitude towards life?” as a “global measure to explore how experiences or behaviour in the travel domain influence overall life satisfaction”. Responses scaled from “indifferent” to “happy and interested”. The results reinforce the evidence that active travel positively impacts life satisfaction.

In 2019, Brainard and colleagues (Brainard et al., 2019) published the results of a cross-sectional analysis of data from the Adult Lives Survey 2016/2017 (Ipsos Mori, 2018) of people aged 16-104 years living in England. Active travelling was investigated by asking how many days, in the last 28 days, they did “…any walking (or cycling) primarily to get from place to place (e.g., walking to work), rather than for health or recreation” of a minimum 10 minutes duration and the trip duration. Part of the sample received questions about “mood traits”. These questions investigated happiness (affect), anxiety, eudaimonia (sense of worth), life satisfaction (“On a scale of 0–10, where 0 is not at all satisfied and ten is completely satisfied, overall, how satisfied are you with your life nowadays?”), and self-efficacy (personal effectiveness) using the items previously listed by the ONS analysis (Office for National Statistics, 2014). The results showed that life satisfaction was not significantly related to active travel, either walking or cycling.

In 2020, Clark and colleagues (Clark *et al.* 2020) used data from six waves of the Understanding Society (2009/10 – 2014/15; UKHLS, previously titled BHPS; University of Essex, 2016) to analyse the impact of commuting in terms of time (duration) and mode on subjective well-being, measuring multiple outcomes. Life satisfaction (“How dissatisfied or satisfied are you with your life overall?”) did not report notable differences in commute time/mode associations. However, it might be interesting to notice that the authors also explored *Satisfaction with leisure-time availability* (“How dissatisfied or satisfied are you with the amount of leisure time you have?”), discovering that only walking to work was associated with increased leisure-time satisfaction. Authors suggested that commuting could negatively impact overall life satisfaction if other benefits (job satisfaction or leisure-time availability) are compromised.

In 2021, Wang and colleagues (Wang *et al.* 2021) analysed cross-sectional data from two waves of the China Labor-force Dynamics Survey (CLDS, 2014 – 2016). Moreover, they called this methodology *quasi-longitudinal*. The study had two main parts: the first related to the impact of commuting behaviour (and haze pollution) on people’s life satisfaction, and the second on the moderating effect of haze pollution on the relationship between commute behaviour and life satisfaction. First, interviewers investigated life satisfaction: “How satisfied are you with your life on the whole?” and commuting behaviour referred to time and mode. The results showed a significant positive relationship with life satisfaction for active travel more than “transit” (car and public transport).

QUASI-EXPERIMENTAL:

In 2021, Jacob and colleagues (Jacob *et al.* 2021) analysed data from seven waves of the UK Household Longitudinal Survey (UKHLS) from 2009 to 2016. Asking about the travel mode (‘How do you usually get to your place of work?’), the large sample was stratified into ‘treated’ for those who changed commute mode over time and ‘controls’ for those who did not. The primary outcomes were overall health and self-reported satisfaction with health. Specifically, the UKHLS assessed life satisfaction by asking, “How satisfied do you feel with your overall life?”. The main results indicate that mode changes from public transport to active travel are associated with better satisfaction. However, transitions between car and public transport are not accompanied by notable impacts on satisfaction.

6. Travel Satisfaction

Before including travel satisfaction in the scoping review, we wanted proof of its correlation with other mental health outcomes. Previous evidence has shown that travel satisfaction is connected to life satisfaction (Gatersleben & Uzzell, 2007) and emotional well-being(Olsson *et al.* 2013). Hence, the work commute may influence subjective well-being’s cognitive and affective components (Ettema *et al.* 2010). In Sweden, Ettema and colleagues (Ettema *et al.* 2011) developed the Satisfaction with Travel Scale (STS) that embraces the following:

- A cognitive evaluation of the quality of travel
- An affective evaluation of feelings during travel ranging from stressed to relaxed
- An affective evaluation of feelings during travel ranging from bored to excited.

In 2017, Smith (Smith 2017) contributed to the study of the travel-to-work experience by discussing *commute well-being.* Smith defined this concept as a “multi-item measure of how one feels about the commute to work and its associated factors”. The study was conducted in Oregon, USA, with four travel modes: walking, cycling, public transport and car. Survey questions were developed using an adapted version of the STS (Ettema et al., 2010), innovatively adding an indicator of enjoyment. This paper confirmed that Commute Well-Being Scale reliably measures commute satisfaction. The same year, Friman and colleagues (Friman *et al.* 2017) expanded this knowledge by studying the satisfaction with any daily travel (work, school, leisure, shopping trips) and its relationship with life satisfaction (cognitive component) and *emotional well-being* (affective component), again in Sweden. Again, the following measures were used:

- STS to measure travel satisfaction (Ettema et al., 2011)
- Swedish Core Affect Scale to measure emotional well-being (SCAS; Västfjäll et al., 2002)
- Satisfaction with Life Scale to measure life satisfaction (Diener *et al.* 1985).

The results showed that travel satisfaction was positively correlated with both well-being outcomes, directly and indirectly. Therefore, in 2021, Liu and colleagues (Liu *et al.* 2021) enlarged the investigation, including the eudaimonic well-being (sense of purpose/meaning in life), other than the hedonic (pleasure, happiness, satisfaction) in Heze, China. The factors investigated were:

- *Commuting experience,* “the activity of regularly travelling to work” (Collins English Dictionary), excluding social purposes, such as shopping or visiting friends, measured with the Journey Experience Scale (JES, Hickman et al., 2013)
- *Hedonic well-being*, measured with the STS (Ettema et al., 2011)
- *Eudaimonic well-being*, measured using the Flourishing Scale (Diener et al., 2010)

The results indicated that commuting experience was positively associated with hedonia (as previously claimed) and that hedonic and eudaimonic well-being were positively correlated. However, no direct association was found between commuting experience and eudaimonia. The authors concluded that despite commuting experience was not directly related to eudaimonic WB, the two variables may be indirectly associated since they are directly connected to hedonic WB.

To sum up, travel satisfaction is positively correlated to life satisfaction and positive affect, while negatively with negative affect; no significant relationship has been found with eudaimonia. The paragraphs below will explain what is known about active mobility modes and the potential travel satisfaction relationship.

CROSS-SECTIONAL:

In 2014, St-Louis and colleagues (St-Louis *et al.* 2014) investigated the different levels of commuting satisfaction in six modes of transportation (walking, cycling, car, bus, metro, and train) in Montrèal, Canada. The survey asked for the description of participants’ commuting on a typical cold, snowy day and a warm, dry day, specifying the mode used and time spent on the mode. Additionally, the survey asked the respondents to rate their agreement on a scale of 1-5 with *aspects of satisfaction* with a given mode.

- Walking: travel time, comfort, safety from traffic, safety from crime, and unwanted attention.
- Cycling: same as walking + quality of cycling pathways.
- Driving: same as walking + cost.
- Bus, metro, train: travel time, consistency of travel time, comfort, safety from crime, unwanted attention, cost, time to reach the destination, waiting time.

Overall trip satisfaction was derived by the sum of every aspect per each mode expressed as a percentage. They found that walking, cycling, and train users were significantly more satisfied than bus, metro and car users.

In 2017, Smith (Smith 2017) validated the *Commute Well-Being Scale,* adapted from the Satisfaction with Travel Scale (STS; Ettema et al., 2010) and analysed how this outcome varies with four travel modes (walking, cycling, public transport, and car) in Oregon, USA. Participants filled out a web-based survey where they reported commute mode and duration. Additionally, the distance was assessed using ArcGIS to geocode residential and work locations. Cycling to work had the highest commute well-being, followed by walking, relatively unaffected by traffic congestion compared to bus and car users.

The same year, Friman and colleagues (Friman et al., 2017) expanded this knowledge by studying satisfaction with any daily travel (work, school, leisure, shopping trips) in Sweden, using the Satisfaction with Travel Scale (STS; Ettema et al., 2011). Again, active travel had a direct association with travel satisfaction; also, life satisfaction and emotional well-being were directly related to travel satisfaction. Additionally, these results suggested that active travel benefits overgo the travel domain (not accounted for by the satisfaction with travel or the commute well-being alone). Lastly, active travel and car use resulted in more satisfaction than public transport.

In 2019, Singleton (Singleton 2019) continued the study of subjective travel well-being in Oregon, USA. The study investigated the most recent commute from home to work (mode and duration). The author asked, “Are there specific subjective well-being constructs where walking and cycling rate are higher or lower than other modes? Are trip or traveller more predictive of travel subjective well-being ratings?”. The author used the following measures:

- Satisfaction with Travel Scale (Ettema et al., 2011)
- Travel Affect. “First, think about yourself and your most recent commute to work, then indicate how you felt”, followed by ten items from I-PANAS-SF (Thompson 2007) (upset, hostile, alert, ashamed, inspired, nervous, determined, attentive, afraid, active) + ten items from psychology and travel literature (excited, strong, vulnerable, proud, angry, bold, frustrated, timid, calm, stressed). The final model had a four-factor structure of travel PANA (Distress and Fear vs Attentiveness and Enjoyment).
- Travel Eudaimonia. They created new items and questions (view paper). The final model identified four constructs (Security, Autonomy, Confidence, and Health) as eudaimonic concepts associated with travel behaviour.

Walking and cycling reported higher physical and mental health scores, confidence, positive affect, and overall hedonic well-being. Nonetheless, cycling scored the highest in distress and fear and lowest in security. The authors suggested a multidimensional measure of travel well-being, including eudaimonia measures, and that active travel could have vital benefits.

In 2021, Liu and colleagues (Liu et al., 2021) took Singleton’s suggestion and included eudaimonic well-being (sense of purpose/meaning in life), other than hedonic well-being (pleasure, happiness, satisfaction), in studying the commuting experience in Heze, China. They examined the commuting experience (travel behaviours in terms of daily commute mode choice). Travel satisfaction was measured with the Satisfaction with Travel Scale (STS; Ettema et al., 2011). Again, public transport, walking and cycling were reported to be more likely to increase well-being components other than improving the quality of the commuting experience overall.

The same year, Fan and colleagues (Fan *et al.* 2021) published another study on this topic in Beijing, China. The travel characteristics (purpose, mode, duration, perceived trip duration, time of the day, day of the week, arrival flexibility, travel companion, and activities during travelling) were examined through a web-based survey. In addition, the authors used the term “travel happiness” measured with the Satisfaction with Travel Scale (Ettema et al., 2011) and analysed what travel modes could be related to this outcome. Overall, active travel users were reported to be the happiest travellers, followed by car drivers and public transport users. Also, walking had higher travel happiness scores than cycling.

7. Stress

CROSS-SECTIONAL:

In 2011, Hansson and colleagues (Hansson *et al.* 2011) measured *Everyday stress* (“Do you feel stressed in your everyday life?”) related to active travelling investigated as commuting mode (How do you usually get to work?”), time (“How long does it take to get to work?”). They found a non-significant association between stress and active commuting. Moreover, they hypothesised a “healthy commuter effect” where car commuters might face more stressful situations, so healthy people are more likely to start commuting actively.

In 2017, Avila-Palencia and colleagues (Avila-Palencia *et al.* 2017) implemented a study to examine the relationship between sole cycling and perceived stress in Barcelona, Spain, as part of the Transportation, Air Pollution and Physical Activities Travel Survey (TAPAS, 2008). The authors measured cycling frequency in days. They also measured people’s propensity to cycle (willingness; “they would consider cycling a mode of transport”). The outcome measure was the Perceived Stress Scale (PSS-4; (Cohen *et al.* 1983). The results showed that bicycle commuters had a significantly lower risk of being stressed than non-bicycle commuters, even after adjusting for confounding individual and environmental factors.

In 2018, Mattisson and colleagues (Mattisson *et al.* 2018) investigated the relationship between commute mode choice and physical and mental health in Scania, Sweden. Their question was if and how people’s travel mode choice is influenced by health status to investigate the “healthy commuter effect” outlined by Hansson and colleagues (2011). Participants were asked, “How do you usually travel to work?”. The authors measured many health status-related aspects: vitality/exhaustion, long-term illness, walking difficulties, obesity and *everyday stress.* Asking “Do you feel stressed in your everyday life?” the results showed a negative association with active travel. Also, public transport users were the ones who reported “stressed often” the most.

In 2020, Sattler and colleagues (Sattler *et al.* 2020) implemented an innovative study. They created a design where the relationship between commute mode and perceived stress was measured up to three days after the end of the trip. This study is part of the ‘Healthy On The way’ (HOTway) study in Graz, Austria. The authors adopted seven statements from the Perceived Stress Questionnaire (PSQ; Levenstein et al., 1993) and the Perceived Stress Scale (PSS-4; Cohen et al., 1983). The scales were filled before the commuting (baseline stress) and after the commuting (referring back to how they felt during the commute; commute stress). The results indicated that active commuting was associated with lower perceived commuting stress than passive commuters (car, motorbike, public transport).

LONGITUDINAL:

In 2018, the authors working on the Physical Activity through Sustainable Transport Approaches project (PASTA project; Gerike et al., 2016) analysed the data collected between 2014 and 2016 to evaluate the association between transport modes (“How often do you currently use each of the following methods of travel to get to and from places?”) and health and social contact measures in seven European cities (Avila-Palencia et al., 2018). The authors evaluated the impact of single and multiple transport modes on the outcomes. Perceived stress, vitality and mental health were components of overall mental health outcomes (Avila-Palencia *et al.* 2018). Stress levels were measured using the Perceived Stress Scale (PSS-4; Cohen et al., 1983) as a mental health outcome. They found that bicycle use was the transport mode associated with the lowest stress levels.

8. Depressive symptoms

In 2020, Marques and colleagues published a review on the relationship between active commuting and depression among adults (Marques *et al.* 2020). The authors identified seven articles published up to 2019; only two showed that those with higher levels of active travelling were less likely to develop depressive symptoms. One of these two papers is described below (Knott *et al.* 2018). In the other five studies, no significant relationship was found. Also, there was no experimental evidence.

CROSS-SECTIONAL:

In 2010, Bergland and colleagues (Bergland *et al.* 2010) investigated a sample of older adults (age 55-79) to understand the relationship between outdoor walking ability (Telephone Survey ‘What is the longest distance you can walk without a pause?’; “active” if walking≥1km) and their self-esteem, coping and health status. This study was part of the NorLAG (Norway; Solem, 2003). Depression was measured with the Center for Epidemiologic Studies Scale (CES-D; Radloff, 1977), included in the overall health status. However, depression scores did not have a significant relationship with outdoor mobility.

In 2021, Muñiz and colleagues (Muñiz *et al.* 2021) used data from the 2014 European Health Survey in Spain, EHSS-2014 - INE (Istituta Nacional de Estadística [Spanish Statistical Office], 2015). People were categorised as active travellers if they responded at least three times per week to the question, “How many days do you walk or use the bike to make trips?”. Depressive symptoms were measured by asking the participants about the presence of symptoms or diagnoses of depression in the previous 12 months. The results showed that travel-related physical activity reduced the likelihood of depression.

LONGITUDINAL:

In 2018, Knott and colleagues (Knott et al., 2018 analysed data from the UK Biobank collected between 2006 and 2016 (Biobank 2007). Active travel was assessed in terms of mode, frequency and distance. The severity of depressive symptoms was determined through the two-item Patient Health Questionnaire (PHQ-2, Kroenke et al., 2003), showing that non-clinical commuters aged 40-75 reported less severe symptoms at follow-up after changing inactive to active mode compared to those who did not.

In 2019, Yang and colleagues (Yang *et al.* 2019) examined the impact of daily transport on depression using an agent-based model (ABM, Nianogo & Arah, 2015; data from US Census 2017; 65-85 years old sample). Depression was operationalised using the Center for Epidemiologic Studies Depression Scale-8 (CESDS-8; West et al., 2014). The authors investigated the impact of three scenarios: improving walkability and safety in the neighbourhood, decreasing bus fares and waiting time, and adding new lines and bus stops. The study concluded that improving walkability and safety had almost no effect on depression prevalence while enhancing public transport services (second and third scenarios) would be the most effective intervention to reduce depression prevalence.

9. Anxiety

CROSS-SECTIONAL:

In 2010, Bergland and colleagues (Bergland et al., 2010) investigated a sample of older adults (age 55-79) to understand the relationship between outdoor walking ability (Telephone Survey ‘What is the longest distance you can walk without a pause?’; “active” if walking≥1km) and their self-esteem, coping and health status. This study was part of the NorLAG (Norway; Solem, 2003). Anxiety was included in the overall health status and measured with the Hopkins Symptoms Check List (HSCL; Parloff et al., 1954). However, they found no relationship between anxiety scores and outdoor mobility.

In 2014, the Office for National Statistics (Office for National Statistics, UK) examined the relationship between time and mode used to commute to work (“Do you work from home?”; “What is the travel time in minutes from home to work, one way?”) and ‘personal well-being’, defined as composed of happiness, anxiety, life satisfaction and worthiness. The survey asked, “Overall, how anxious did you feel yesterday?”. In general, commuters to work were more anxious than non-commuters. Moreover, respondents who travelled by public transport (specifically trains) had the highest anxiety levels. Other commute modes’ impacts were mediated by commuting time: cycling and walking for more than 15 minutes was associated with higher anxiety levels.

In 2019, Brainard and colleagues (Brainard et al., 2019) published the results of a cross-sectional analysis of data from the Adult Lives Survey 2016/2017 (Ipsos Mori, 2018) of people aged 16-104 years living in England. Active travelling was investigated by asking how many days, in the last 28 days, they did “…any walking (or cycling) primarily to get from place to place (e.g., walking to work), rather than for health or recreation” of a minimum 10 minutes duration and the trip duration. Moreover, part of the sample received questions about “mood traits”. These questions investigated happiness (affect), anxiety, sense of worth (eudaimonia), life satisfaction, and personal effectiveness (self-efficacy) using the same items previously listed by the ONS paper (Office for National Statistics, 2014). The results showed that anxiety was not significantly related to active travel, either walking or cycling.

10. Loneliness

CROSS-SECTIONAL:

In 2012, Smith (Smith, 2012) measured loneliness levels using the UCLA Loneliness Scale - Version 3 (Austin, 1983; Russell, 1996). It was an interpretative phenomenological analysis. Travel habits were discussed through narrative data using the “Daily Life Interview”. The author found that loneliness increased due to “disrupted engagement with others”, and one of the reasons was reduced mobility: some participants (aged 74-98) stopped driving a car, and the loss of transportation interfered with social connections maintenance. Since the primary coping strategy acted by the sample was reaching others to recover social connections, Smith suggested that it is vital to be mindful of these people’s transportation needs.

LONGITUDINAL:

In 2018, the authors working on the Physical Activity through Sustainable Transport Approaches project (PASTA project, Gerike et al., 2016) analysed the data collected between 2014 and 2016 to evaluate the association between transport modes (“How often do you currently use each of the following methods of travel to get to and from places?”) and health and social contact measures in seven European cities (Avila-Palencia et al., 2018). In addition, the authors evaluated the impact of single and multiple transport modes on the outcomes. The UCLA Loneliness Scale (Russell, 1996) was one of the social contact measures used. The results highlighted that cycling in multimodal trips and car use were associated with lower loneliness feelings than other transport modes. On the other hand, motorbike, e-bike, and public transport modes had non-significant and inconclusive results.

11. Social Support

CROSS-SECTIONAL:

In 2011, Smith used the “Daily Life Interview” (Smith 2012) to collect information about the sample’s ongoing connections with friends and family. Travel habits were also discussed through narrative data using the “Daily Life Interview” (Smith, 2012). It was an interpretative phenomenological analysis. The author found that social contacts decreased due to “disrupted engagement with others”, and one of the reasons was reduced mobility: some participants (aged 74-98) stopped driving a car, and the loss of transportation interfered with social connections maintenance. Since the primary coping strategy acted by the sample was reaching others to recover social connections, Smith suggested that it is vital to be mindful of these people’s transportation needs.

LONGITUDINAL:

In 2018, Avila-Palencia and colleagues (Avila-Palencia, et al., 2018) measured social contacts about active travel modes for the first time. The subjects were asked, “How often do you currently use each of the following methods of travel to get to and from places?”. In addition, the authors evaluated the impact of single and multiple transport modes on the outcomes. The social contact measure was the single item, “How often do you have contact with your friends and/or family?”. The results showed a significant positive association between walking in multimodal trips and increased social contacts, thus more frequent contact with friends/family for those who used walking as a means of transport, compared to other transport modes.

12. Self-Efficacy

CROSS-SECTIONAL:

In 2010, Bergland and colleagues (Bergland et al., 2010) studied outdoor walking mobility as the instrumental ability to move by foot for any trip outside the home (Telephone Survey ‘What is the longest distance you can walk without a pause?’; “active” if walking for more than 1 km) and its relationship with what they called “coping”. This outcome was measured using the Personal Mastery Scale, which measures “one aspect of psychological coping resources, Mastery, or Self-Efficacy” (Pearlin and Schooler 1978). The results showed better self-efficacy scores for those with good outdoor walking mobility (walking for 1km or more).

In 2010, Molina-García and colleagues (Molina-García *et al.* 2010) investigated the relationship between commuting to university (‘How often do you use each of the following ways to go to and from the university?’) and psychosocial (and environmental) factors in a sample of 518 students. One of the psychosocial constructs was physical self-efficacy, rated through the Physical Self-Efficacy subscale of the Perceived Physical Activity (PPA) scale (Ryckman *et al.* 1982). The analysis showed a significant correlation between active commuting to university and self-efficacy.

In 2016, Bélanger-Gravel and colleagues (Bélanger-Gravel *et al.* 2016) analysed the results from two cross-sectional population-based surveys, T1 (2009) and T2 (2010). The measures came from a natural experiment in Montréal, Canada, where a public bike-sharing program was implemented (BIXI – Bicycle-taXI). The participants were asked, “Have you ever used PBSP?”. Self-efficacy in using the program was assessed with a 1-item question “How confident are you in your capability of using BIXI if you chose to do so?”. The authors observed increased self-efficacy levels; consequently, they affirmed that implementing a similar program increases active travelling, which is significantly connected to higher self-efficacy.

In 2019, Brainard and colleagues (Brainard et al., 2019) published the results of a cross-sectional analysis of data from the Adult Lives Survey 2016/2017 of people aged 16-104 years living in England. First, active travelling was investigated by asking how many days, in the last 28 days, they did “…any walking (or cycling) primarily to get from place to place (e.g., walking to work), rather than for health or recreation” of a minimum 10 minutes duration and the trip duration. Moreover, part of the sample received questions about “mood traits”. These questions investigated happiness (affect), anxiety, sense of worth (eudaimonia), life satisfaction, and personal effectiveness (self-efficacy) using the same items previously listed by the ONS paper (Office for National Statistics, 2014). The results showed that self-efficacy (“To what extent do you agree with the statement: I can achieve most of the goals I set myself?” from (1) Strongly agree to (5) Strongly disagree) was the only “mood trait” significantly connected with walking; none was related to cycling.

13. Sleep

CROSS-SECTIONAL:

In 2011, Hansson and colleagues (Hansson et al., 2011) measured commuting to work mode (How do you usually get to work?”), time (“How long does it take to get to work?”), concerning many health outcomes. One was perceived sleep quality, measured with the item “Do you think you get enough sleep to feel rested?”. The two variables showed no significant relationship; however, they stated that it was close to being significant (p=0.06). Although they observed no significant association between commuting time and mode and any health outcome, they described the “healthy commuter effect”, so people facing illness and stress might be less prone to start or continue commuting actively.

In 2018, Foley and colleagues (Foley *et al.* 2018) examined data from the 2014/2015 United Kingdom Harmonised European Time Use Survey (Gershuny and Sullivan 2017). The survey provided a “time-use” diary with a “Where” section where people could indicate if they had travelled on foot or by bike. Sleep was measured with minutes/day, and the scores showed that travelling on foot or by bike was associated with lower sleep duration. However, active travel also reduced screen time, a positive result. Since shorter or longer sleep duration could be detrimental to people’s health, interpreting these results was difficult for the authors without considering baseline health levels.

In 2018, Vancampfort and colleagues (Vancampfort *et al.* 2018) analysed data from the World Health Organization’s Study on Global Ageing and adult health (SAGE; Kowal et al., 2012) (China, Ghana, India, Mexico, Russia, South Africa). Active travel, in terms of walking and cycling, was assessed through the GPAQ (Bull *et al.* 2009), asking individuals about the usual way to “travel to and from places (e.g., getting to work, to shopping, to the market, to place of worship etc.)”. Active travel time was calculated according to how many days per week (frequency) and how long per day (duration) people would walk or bicycle continuously for at least 10 minutes to get to and from places. In addition, the 1-item question “Overall in the last 30 days, how much of a problem did you have with sleeping, such as falling asleep, waking up frequently during the night or waking up too early in the morning?” measured sleep quality. Results showed that people with the lowest active travel levels also had worse sleep quality. Furthermore, the authors highlighted that sleep was not a significant mediator in the relationship between low levels of active travel and chronic physical conditions.

14. Vitality, exhaustion, fatigue, and strain

CROSS-SECTIONAL:

In 2011, Hansson and colleagues (Hansson et al., 2011) measured commuting to work mode (“How do you usually get to work?”), time (“How long does it take to get to work?”) and *exhaustion* in terms of “low vitality” using the Vitality scale from the Sweden version of the SF-36 (Sullivan *et al.* 1995). They found that exhaustion was one of the health outcomes most clearly associated with commuting; specifically, active travelling had a negative relationship with exhaustion, so people who walk or cycle would report higher vitality scores. However, this relationship was only “close to be statistically significant” (p= 0.30).

In 2018, another team of experts repeated these measures in Sweden (Mattisson, et al., 2018), reporting a non-significant relationship between active transport modes and vitality levels.

In 2020, Clark and colleagues (Clark et al., 2020) used data from six waves of the Understanding Society (2009/10 – 2014/15; UKHLS, previously titled BHPS; University of Essex, 2016) to analyse the impact of commuting in terms of time (duration) and mode on subjective well-being, measuring multiple outcomes. One was *strain*, measured with the single item “Have you recently (last few weeks) felt constantly under strain?”. Those who walked to work showed lower strain levels than car drivers.

LONGITUDINAL:

In 2018, Avila-Palencia and colleagues (Avila-Palencia et al., 2018) measured social contacts about active travel modes for the first time. The subjects were asked, “How often do you currently use each of the following methods of travel to get to and from places?”. In addition, the authors evaluated the impact of single and multiple transport modes on the outcomes. They measured vitality (SF-36 – Vitality Scale) (Ware et al., 1993) as a mental health outcome and found a significant positive relationship between cycling, walking, both unimodal and multimodal trips, and vitality scores.

15. Self-reported health

CROSS-SECTIONAL:

In 2010, Bergland and colleagues (Bergland et al., 2010) studied outdoor walking mobility as the instrumental ability to move by foot for any trip outside the home (Telephone Survey ‘What is the longest distance you can walk without a pause?’; “active” if walking for more than 1 km) and its relationship with health status. One outcome contributing to the overall health status was *self-rated* or *self-reported health* and examined by asking, “How do you judge your own general state of health?”. The authors found that being able to walk for at least 1 km or more had significantly higher scores in self-reported health. Furthermore, considering the other health outcomes, self-reported health had the most significant association with the dependent variable.

In 2010, Rasciute and Downward (Rasciute and Downward 2010) used data from the Taking Part Survey (since 2005, DCMS, England). The survey collected data about 67 sports activities, recording participation in the last 12 months and the previous four weeks, the frequency, and the duration of each activity. One of them was cycling for utilitarian purposes. The authors were interested in understanding the impact of these sports activities on health and happiness as two different but related constructs (confirmed by the results of their analysis). Therefore, self-reported health was assessed with a single item, “How is your health in general? (very good, good, fair, bad, very bad)”. Both walking and cycling for “utilitarian” or transport purposes had a statistically significant positive relationship on people’s self-reported health.

In 2011, Hansson and colleagues (Hansson et al., 2011) measured commuting to work mode (How do you usually get to work?”), time (“How long does it take to get to work?”), concerning many health outcomes. One was self-reported health, measured with 1 item “How do you feel right now, physically and psychologically, considering your health and your well-being?” (Eriksson *et al.* 2001). They also investigated sickness absence days. Although they observed no significant association between commuting time and mode and any health outcome, they described the “healthy commuter effect”, so people facing illness and stress might be less prone to start or continue commuting actively.

In 2013, Humphreys and colleagues (Humphreys *et al.* 2013) examined the relationship between active commuting (frequency+duration) and physical well-being. The measure used was the Physical Component Score (PCS) from the SF-8 (Ware et al., 2001). The results showed that greater time spent travelling actively was related to higher physical well-being levels. Data came from the Commuting and Health in Cambridge study (Ogilvie et al., 2010).

In 2016, Mason and colleagues (Mason *et al.* 2016) examined the relationship between physical activity domains (household, occupational, active travel, leisure and sport, and family activities) and mental and physical health. The study was conducted in Glasgow, Scotland, as part of the GoWell Research and Learning Programme (2011). They measured physical health using the Physical Component Score (PCS) from the SF-12 (Ware et al., 1996) and the item “In general, would you say your health is good, very good, excellent?”. In addition, the survey asked participants to “Rank 5 PA domains in order of how much you do each of them’”. The results recorded no significant relationship between the PCS scores and any domain of PA. However, people practising active travel were likelier to report their health as good or excellent.

In 2017, Sener and Lee (Sener and Lee 2017) analysed the El Paso Survey data. The survey measured self-reported health levels (“How would you rate your health?”) and discovered a noticeable relationship with life satisfaction and BMI scores, positive with the former and negative with the latter. In addition, the survey investigated the frequency of different transportation modes. The descriptive analysis confirmed that cyclists had the highest levels of self-reported health. Nonetheless, the regression model claimed a non-significant relationship between self-reported health (or BMI) and walking or cycling.

In 2018, Mattisson and colleagues (Mattisson et al., 2018) highlighted the importance of health status in choosing the commute mode. They investigated people’s health with various outcomes, some related to mental health like stress and vitality, others to physical health like long-standing illness (“Do you have a long-standing health condition, discomfort after an accident, disability or other long term health condition?”), walking difficulties (“Are you, due to your current health condition, limited in any of the following activities?” one was taking a walk), and BMI as an indicator of obesity. Participants were asked, “How do you usually travel to work?”. All these measures were self-reported. The authors found a significant negative relationship between active travel modes and all these outcomes, and the correlation with the physical health outcomes was stronger than the one with the mental health factors.

In 2020, Clark and colleagues (Clark et al., 2020) used data from six waves of the Understanding Society (2009/10 – 2014/15; UKHLS, previously titled BHPS; University of Essex, 2016) to analyse the impact of commuting in terms of time (duration) and mode on subjective well-being, measuring multiple outcomes. One of them was self-reported health. The single-question item asked, “In general, would you say your health is? (from poor to excellent)”. They concluded that, compared to car drivers, cyclists had higher self-reported health, while bus users showed lower scores.

In 2021, Muñiz and colleagues (Muñiz et al., 2021) used data from the 2014 European Health Survey in Spain, EHSS-2014 – INE (Istituta Nacional de Estadística [Spanish Statistical Office], 2015). People were categorised as active travellers if they responded at least three times per week to the question, “How many days do you walk or use the bike to make trips?”. Self-perceived health was part of the health status, together with depression. Self-Assessed Health (SAH) was measured by asking participants, “In the last twelve months, would you say your health has been very good, good, fair, bad, or very bad?”. The results showed that travel-related physical activity was significantly related to better self-perceived health.

LONGITUDINAL:

In 2016, Mytton and colleagues (Mytton *et al.* 2016) conducted a longitudinal study built on Humphreys’s cross-sectional evidence (2013). The authors analysed data from the Commuting and Health in Cambridge study (Ogilvie et al., 2010) to explore longitudinal associations of maintenance and changes in active commuting, physical and mental well-being, and sickness absence as health status indicators. The measures used were the Physical Component Score (PCS) from the SF-8 (Ware et al., 2001) and the self-reported frequency and duration of each transportation mode and trip. Commuters maintaining cycling as travel behaviour reported better physical well-being at follow-up - no significant associations between any change in cycling and MCS. Also, no significant associations were observed for walking.

In 2018, Avila-Palencia and colleagues (Avila-Palencia et al., 2018) measured social contacts about active travel modes for the first time. The subjects were asked, “How often do you currently use each of the following methods of travel to get to and from places?”. The authors evaluated the impact of single and multiple transport modes on the outcomes. Self-perceived health was measured by asking, “In general, how would you say your health is?” from the SF-36 (Ware et al., 1992). The authors found that walking and cycling, in both unimodal and multimodal trips, reported better self-perceived health over time; car and public transport users showed poorer levels.

QUASI-EXPERIMENTAL:

In 2021, Jacob and colleagues (Jacob et al., 2021) analysed data from seven Understanding Society - UK Household Longitudinal Study waves from 2009 to 2016. Asking about the travel mode (‘How do you usually get to your place of work?’), the large sample was stratified into ‘treated’ for those who changed commute mode and ‘controls’ for those who did not. Specifically, SF-12 (Ware et al., 1996) was the health measure summarising mental (SF12-MCS) and physical (SF12-PCS) scores. The main results indicate a significant increase in physical and mental health for commuters who switch from car to active travel, especially women. Conversely, a decline in reported physical health is registered for both sexes when changing from active transport modes to car driving. Notable impacts on health or satisfaction did not accompany car and public transport changes.

Supplementary Appendix A References

Aarts, H, Verplanken, B, and Van Knippenberg, A (1997) Habit and information use in travel mode choices. *Acta Psychologica*, **96**(1–2), 1–14. doi:10.1016/S0001-6918(97)00008-5.

Austin, BA (1983) Factorial Structure of the UCLA Loneliness Scale. *Http://Dx.Doi.Org/10.2466/Pr0.1983.53.3.883*, **53**(3 I), 883–889. doi:10.2466/PR0.1983.53.3.883.

Avila-Palencia, I, de Nazelle, A, Cole-Hunter, T, … Nieuwenhuijsen, MJ (2017) The relationship between bicycle commuting and perceived stress: a cross-sectional study. *BMJ Open*, **7**(6), e013542. doi:10.1136/bmjopen-2016-013542.

Avila-Palencia, I, Int Panis, L, Dons, E, … Nieuwenhuijsen, MJ (2018) The effects of transport mode use on self-perceived health, mental health, and social contact measures: A cross-sectional and longitudinal study. *Environment International*, **120**, 199–206. doi:10.1016/j.envint.2018.08.002.

Baecke, JA, Burema, J, and Frijters, JE (1982) A short questionnaire for the measurement of habitual physical activity in epidemiological studies. *The American Journal of Clinical Nutrition*, **36**(5), 936–942.

Baker, G, Gray, SR, Wright, A, … Mutrie, N (2008) The effect of a pedometer-based community walking intervention “Walking for Wellbeing in the West” on physical activity levels and health outcomes: A 12-week randomized controlled trial. *International Journal of Behavioral Nutrition and Physical Activity*, **5**. doi:10.1186/1479-5868-5-44.

Bélanger-Gravel, A, Gauvin, L, Fuller, D, and Drouin, L (2016) Association of implementation of a public bicycle share program with intention and self-efficacy: The moderating role of socioeconomic status. *Journal of Health Psychology*, **21**(6), 944–953. doi:10.1177/1359105314542820.

Bergland, A, Thorsen, K, and Loland, NW (2010) The relationship between coping, self-esteem and health on outdoor walking ability among older adults in Norway. *Ageing and Society*, **30**(6), 949–963. doi:10.1017/S0144686X1000022X.

Berwick, DM, Murphy, JM, Goldman, PA, Ware, JE, Barsky, AJ, and Weinstein, MC (1991) Performance of a Five-Item Mental Health Screening Test., **29**(2), 169–176.

Biobank, UK (2007) *2007. Protocol for a Large-Scale Prospective Epidemiological Resource [Internet].*, Stockport, UK.

Brainard, J, Cooke, R, Lane, K, and Salter, C (2019) Age, sex and other correlates with active travel walking and cycling in England: Analysis of responses to the Active Lives Survey 2016/17. *Preventive Medicine*, **123**, 225–231. doi:10.1016/j.ypmed.2019.03.043.

Bull, FC, Maslin, TS, and Armstrong, T (2009) Global Physical Activity Questionnaire (GPAQ): Nine Country Reliability and Validity Study. *Journal of Physical Activity and Health*, **6**(6), 790–804. doi:10.1123/jpah.6.6.790.

Bullinger, M (1995) German translation and psychometric testing of the SF-36 Health Survey: Preliminary results from the IQOLA project. *Social Science and Medicine*, **41**(10), 1359–1366. doi:10.1016/0277-9536(95)00115-N.

Burger M., and Veenhoven, R (2016) *The Happiness Indicator: Combination of a self-help website and a scientific follow-up study. Prospectus 2016*, Rotterdam: Erasmus University Rotterdam. Retrieved from http://www.eur.nl/ehero/gelukswijzer

Cerin, E, Leslie, E, Sugiyama, T, and Owen, N (2009) Associations of multiple physical activity domains with mental well-being. *Mental Health and Physical Activity*, **2**(2), 55–64. doi:10.1016/j.mhpa.2009.09.004.

Chng, S, White, M, Abraham, C, and Skippon, S (2016) Commuting and wellbeing in London: The roles of commute mode and local public transport connectivity. *Preventive Medicine*, **88**, 182–188. doi:10.1016/j.ypmed.2016.04.014.

Clark, B, Chatterjee, K, Martin, A, and Davis, A (2020) How commuting affects subjective wellbeing. *Transportation*, **47**(6), 2777–2805. doi:10.1007/s11116-019-09983-9.

Cobbold, A, Standen, C, Shepherd, L, Greaves, S, and Crane, M (2022) Multimodal trips, quality of life and wellbeing: An exploratory analysis. *Journal of Transport and Health*, **24**. doi:10.1016/j.jth.2022.101330.

Cohen, S, Kamarck, T, and Mermelstein, R (1983) A global measure of perceived stress. *Journal of Health and Social Behavior*, **24**(4), 385–396. doi:10.2307/2136404.

Craig, CL, Marshall, AL, Sjo¨stro¨m, M, … Oja, P (2003) International Physical Activity Questionnaire: 12-Country Reliability and Validity. *Med. Sci. Sports Exerc*, **35**(8), 1381–1395. doi:10.1249/01.MSS.0000078924.61453.FB.

De Geus, B, Van Hoof, E, Aerts, I, and Meeusen, R (2008) Cycling to work: Influence on indexes of health in untrained men and women in Flanders. Coronary heart disease and quality of life. *Scandinavian Journal of Medicine and Science in Sports*, **18**(4), 498–510. doi:10.1111/j.1600-0838.2007.00729.x.

De Vos, J (2018) Do people travel with their preferred travel mode? Analysing the extent of travel mode dissonance and its effect on travel satisfaction. *Transportation Research Part A: Policy and Practice*, **117**, 261–274. doi:10.1016/j.tra.2018.08.034.

Diener, E, Emmons, RA, Larsem, RJ, and Griffin, S (1985) The Satisfaction With Life Scale. *Journal of Personality Assessment*, **49**(1), 71–75. doi:10.1207/S15327752JPA4901_13.

Diener, E, Wirtz, D, Biswas-Diener, R, … Oishi, S (2009) New Measures of Well-Being., 247–266. doi:10.1007/978-90-481-2354-4_12.

Diener, E, Wirtz, D, Tov, W, … Biswas-Diener, R (2010) New well-being measures: Short scales to assess flourishing and positive and negative feelings. *Social Indicators Research*, **97**(2), 143–156. doi:10.1007/S11205-009-9493-Y.

Eriksson, I, Undén, AL, and Elofsson, S (2001) Self-rated health. Comparisons between three different measures. Results from a population study. *International Journal of Epidemiology*, **30**(2), 326–333. doi:10.1093/IJE/30.2.326.

Ettema, D, Gärling, T, Eriksson, L, Friman, M, Olsson, LE, and Fujii, S (2011) Satisfaction with travel and subjective well-being: Development and test of a measurement tool. *Transportation Research Part F: Traffic Psychology and Behaviour*, **14**(3), 167–175. doi:10.1016/J.TRF.2010.11.002.

Ettema, D, Gärling, T, Olsson, LE, and Friman, M (2010) Out-of-home activities, daily travel, and subjective well-being. *Transportation Research Part A: Policy and Practice*, **44**(9), 723–732. doi:10.1016/j.tra.2010.07.005.

Fan, A, Chen, X, and Zhang, X (2021) What Factors Contribute to Higher Travel Happiness? Evidence from Beijing, China. *Journal of Advanced Transportation*, **2021**. doi:10.1155/2021/8861841.

Fitzsimons, CF, Baker, G, Wright, A, … Mutrie, N (2008) The “Walking for Wellbeing in the West” randomised controlled trial of a pedometer-based walking programme in combination with physical activity consultation with 12 month follow-up: Rationale and study design. *BMC Public Health*, **8**. doi:10.1186/1471-2458-8-259.

Fleck, MP, Louzada, S, Xavier, M, … Pinzon, V (2000) Aplicação da versão em português do instrumento abreviado de avaliação da quali-dade de vida “WHOQOL-bref” Application of the Portuguese version of the abbreviated instrument of quality life WHOQOL-bref. *Revista de Saúde Pública*, **32**(2), 178–183.

Fleming, JS, and Courtney, BE (1984) The dimensionality of self-esteem: II. Hierarchical facet model for revised measurement scales. *Journal of Personality and Social Psychlogy*, **46**(2), 404.

Foley, L, Dumuid, D, Atkin, AJ, Olds, T, and Ogilvie, D (2018) Patterns of health behaviour associated with active travel: A compositional data analysis. *International Journal of Behavioral Nutrition and Physical Activity*, **15**(1). doi:10.1186/s12966-018-0662-8.

Friman, M, Gärling, T, Ettema, D, and Olsson, LE (2017) How does travel affect emotional well-being and life satisfaction? *Transportation Research Part A: Policy and Practice*, **106**, 170–180. doi:10.1016/j.tra.2017.09.024.

Gatersleben, B, and Uzzell, D (2007) Affective appraisals of the daily commute: Comparing perceptions of drivers, cyclists, walkers, and users of public transport. *Environment and Behavior*, **39**(3), 416–431. doi:10.1177/0013916506294032.

Gerike, R, De Nazelle, A, Nieuwenhuijsen, M, … Götschi, T (2016) Physical Activity through Sustainable Transport Approaches (PASTA): A study protocol for a multicentre project. *BMJ Open*, **6**(1). doi:10.1136/bmjopen-2015-009924.

Gershuny, J, and Sullivan, O (2017) United kingdom time use survey, 2014-2015. centre for time use research, university of oxford. Retrieved from http://doi. org/10.5255/UKDA-SN-8128-1

Glasgow, TE, Le, HTK, Scott Geller, E, Fan, Y, and Hankey, S (2019) How transport modes, the built and natural environments, and activities influence mood: A GPS smartphone app study. *Journal of Environmental Psychology*, **66**. doi:10.1016/j.jenvp.2019.101345.

Glasgow, TE, Scott Geller, E, Le B, TK, and Hankey, S (2018) Travel mood scale: Development and validation of a survey to measure mood during transportation. doi:10.1016/j.trf.2018.09.014.

Goldberg, D (1978) *Manual of the general health questionnaire*, Nfer Nelson.

Goldberg, DP, Gater, R, Sartorius, N, … Rutter, C (1997) The validity of two versions of the GHQ in the WHO study of mental illness in general health care. *Psychological Medicine*, **27**(1), 191–197. doi:10.1017/S0033291796004242.

Gómez, LF, Moreno, J, Gómez, OL, Carvajal, R, and Parra, DC (2013) Physical activity and health-related quality of life among adult women in Cali, Colombia: a cross-sectional study. *Quality of Life Research*, **22**(9), 2351–2358. doi:10.1007/s11136-013-0378-9.

GoWell (2010) GoWell: Progress for People and Places: Monitoring change in Glasgow’s communities. Evidence from the GoWell Surbeys 2006 and 2008. Retrieved from https://scholar-google-com.ezproxy.unibo.it/scholar_lookup?title=Progress%20for%20people%20and%20places%3A%20Monitoring%20change%20in%20Glasgow%27s%20communities.%20Evidence%20from%20the%20GoWell%20surveys%202006%20and%202008&author=GoWell&publication_year=2010

Guralnik, JM, Lacroix, AZ, Abbott, RD, … Wallace, RB (1993) Maintaining mobility in late life. I. Demographic characteristics and chronic conditions. *American Journal of Epidemiology*, **137**(8), 845–857. doi:10.1093/oxfordjournals.aje.a116746.

Hansson, E, Mattisson, K, Bjork, J, Ostergren, P-O, and Jakobsson, K (2011) Relationship between commuting and health outcomes in a cross-sectional population survey in southern Sweden. *Bmc Public Health*, **11**, 834. doi:10.1186/1471-2458-11-834.

Hays, RD, and Reeve, BB (2010) *Epidemiology and Demography in Public Health*, San Diego, CA: Academic Press.

Heikkinen, E (1998) Background, Design, and Methods of the Evergreen Project. *Journal of Aging & Physical Activity*, **6**(2).

Hickman, R, Hall, P, and Banister, D (2013) Planning more for sustainable mobility. *Journal of Transport Geography*, **33**, 210–219. doi:10.1016/j.jtrangeo.2013.07.004.

Humphreys, DK, Goodman, A, and Ogilvie, D (2013) Associations between active commuting and physical and mental wellbeing. *Preventive Medicine*, **57**(2), 135–139. doi:10.1016/j.ypmed.2013.04.008.

INE (Istituta Nacional de Estadística) [Spanish Statistical Office] (2015) *Encuesta europea de salud en España 2014 (EESE-2014) [European Health Survey in Spain (EHSS)]*.

Ipsos Mori (2018) *Active Lives Survey Y2 Data User Guide*, London.

Jacob, N, Munford, L, Rice, N, and Roberts, J (2021) Does commuting mode choice impact health? *Health Economics (United Kingdom)*, **30**(2), 207–230. doi:10.1002/hec.4184.

Jurakić, D, Pedišić, Ž, and Greblo, Z (2010) Physical activity in different domains and health-related quality of life: a population-based study. *Quality of Life Research*, **19**(9), 1303–1309. doi:10.1007/s11136-010-9705-6.

Kahneman, D, Krueger, AB, Schkade, DA, Schwarz, N, and Stone, AA (2004) A survey method for characterizing daily life experience: The day reconstruction method. *Science*, **306**(5702), 1776–1780. doi:10.1126/SCIENCE.1103572.

Kaplan, S, Wrzesinska, DK, and Prato, CG (2019) Psychosocial benefits and positive mood related to habitual bicycle use. *Transportation Research Part F: Traffic Psychology and Behaviour*, **64**, 342–352. doi:10.1016/j.trf.2019.05.018.

Knott, CS, Panter, J, Foley, L, and Ogilvie, D (2018) Changes in the mode of travel to work and the severity of depressive symptoms: a longitudinal analysis of UK Biobank. *Preventive Medicine*, **112**, 61–69. doi:10.1016/j.ypmed.2018.03.018.

Kowal, P, Chatterji, S, Naidoo, N, … Newell, ML (2012) Data resource profile: the World Health Organization Study on global AGEing and adult health (SAGE). *International Journal of Epidemiology*, **41**(6), 1639–1649. doi:10.1093/IJE/DYS210.

Kroenke, K, Spitzer, RL, and Williams, JBW (2003) The Patient Health Questionnaire-2: validity of a two-item depression screener. *Medical Care*, 1284–1292.

Kroesen, M, and De Vos, J (2020) Does active travel make people healthier, or are healthy people more inclined to travel actively? *Journal of Transport and Health*, **16**. doi:10.1016/j.jth.2020.100844.

Lampinen, P, Heikkinen, RL, Kauppinen, M, and Heikkinen, E (2006) Activity as a predictor of mental well-being among older adults. *Aging and Mental Health*, **10**(5), 454–466. doi:10.1080/13607860600640962.

Lancée, S, Veenhoven, R, and Burger, M (2017) Mood during commute in the Netherlands: What way of travel feels best for what kind of people? *Transportation Research Part A: Policy and Practice*, **104**, 195–208. doi:10.1016/j.tra.2017.04.025.

Levenstein, S, Prantera, C, Varvo, V, … Andreoli, A (1993) Development of the perceived stress questionnaire: A new tool for psychosomatic research. *Journal of Psychosomatic Research*, **37**(1), 19–32. doi:10.1016/0022-3999(93)90120-5.

Lira, BM, and Paez, A (2021) Do drivers dream of walking? An investigation of travel mode dissonance from the perspective of affective values. *Journal of Transport and Health*, **20**. doi:10.1016/j.jth.2021.101015.

Liu, Q, Chen, CL, and Cao, M (2021) Exploring the relationship between the commuting experience and hedonic and eudaimonic well-being. *Transportation Research Part D: Transport and Environment*, **99**. doi:10.1016/j.trd.2021.103026.

Loidl, M, Stutz, P, Fernandez Lapuente de Battre, MD, … Niederseer, D (2020) Merging self-reported with technically sensed data for tracking mobility behavior in a naturalistic intervention study. Insights from the GISMO study. *Scandinavian Journal of Medicine and Science in Sports*, **30**(S1), 41–49. doi:10.1111/SMS.13636.

Lowther, M, Mutrie, N, Loughlan, C, and Mcfarlane, C (1999) Development of a Scottish physical activity questionnaire: a tool for use in physical activity interventions. *Br J Sports Med*, **33**, 244–249. doi:10.1136/bjsm.33.4.244.

Marques, A, Peralta, M, Henriques-Neto, D, Frasquilho, D, Gouveira, ER, and Gomez-Baya, D (2020) Active Commuting and Depression Symptoms in Adults: A Systematic Review. *International Journal of Environmental Research and Public Health*, **17**(3), 1041. doi:10.3390/ijerph17031041.

Martin, A, Goryakin, Y, and Suhrcke, M (2014) Does active commuting improve psychological wellbeing? Longitudinal evidence from eighteen waves of the British Household Panel Survey. *Preventive Medicine*, **69**, 296–303. doi:10.1016/j.ypmed.2014.08.023.

Mason, P, Curl, A, and Kearns, A (2016) Domains and levels of physical activity are linked to adult mental health and wellbeing in deprived neighbourhoods: A cross-sectional study. *Mental Health and Physical Activity*, **11**, 19–28. doi:10.1016/j.mhpa.2016.07.001.

Mattisson, K, Idris, AO, Cromley, E, Håkansson, C, Östergren, P-O, and Jakobsson, K (2018) Modelling the association between health indicators and commute mode choice: A cross-sectional study in southern Sweden. *Journal of Transport & Health*, **11**, 110–121. doi:10.1016/j.jth.2018.10.012.

McCarthy, S, and Habib, MA (2018) Investigation of life satisfaction, travel, built environment and attitudes. *Journal of Transport and Health*, **11**, 15–24. doi:10.1016/j.jth.2018.09.007.

Molina-García, J, Castillo, I, and Sallis, JF (2010) Psychosocial and environmental correlates of active commuting for university students. *Preventive Medicine: An International Journal Devoted to Practice and Theory*, **51**(2), 136–138. doi:10.1016/j.ypmed.2010.05.009.

Morris, EA, and Guerra, E (2015) Mood and mode: does how we travel affect how we feel? *Transportation*, **42**(1), 25–43. doi:10.1007/s11116-014-9521-x.

Muñiz, C, Rodríguez, P, and Suárez, MJ (2021) An insight into physical activity across domains: implications for depression and perceived health. *European Sport Management Quarterly*. doi:10.1080/16184742.2021.1890801.

Murphy, B, Herrman, H, Hawthorne, G, Pinzone, T, and Evert, H (2000) *Australiano WHOQoL Instruments: User’s Manual and Interpretation Guide.*

Mutrie, N, Carney, C, Blamey, A, Crawford, F, Aitchison, T, and Whitelaw, A (2002) “Walk in to Work Out”: A randomised controlled trial of a self help intervention to promote active commuting. *Journal of Epidemiology and Community Health*, **56**(6), 407–412. doi:10.1136/jech.56.6.407.

Mutrie, N, Cerney, C, Blamey, A, Whitelaw, A, Crawford, F, and Aitchison, T (2000) PART II: INTERDISCIPLINARY. In *Journal of Sports Sciences*, Vol. 18, Informa UK Limited, , 12–21. doi:10.1080/026404100365252.

Mytton, OT, Panter, J, and Ogilvie, D (2016) Longitudinal associations of active commuting with wellbeing and sickness absence. *Preventive Medicine*, **84**, 19–26. doi:10.1016/j.ypmed.2015.12.010.

Neumeier, LM, Loidl, M, Reich, B, … Niederseer, D (2020) Effects of active commuting on health-related quality of life and sickness-related absence. *Scandinavian Journal of Medicine & Science in Sports*, **30 Suppl 1**, 31–40. doi:10.1111/sms.13667.

Nianogo, RA, and Arah, OA (2015) Agent-Based Modeling of Noncommunicable Diseases: A Systematic Review. *American Journal of Public Health*, **105**(3), e20. doi:10.2105/AJPH.2014.302426.

OECD (2010) Sickness, disability and work: Breaking the barriers: A synthesis of findings across OECD countries. Retrieved from https://doi.org/10.1787/9789264088856-en

OECD (2013, March 20) OECD Guidelines on Measuring Subjective Well-being. doi:10.1787/9789264191655-EN.

Office for National Statistics (2014) *Commuting and Personal Well-being, 2014*, London: The National Archives. Retrieved from https://webarchive.nationalarchives.gov.uk/ukgwa/20160105231823/http://www.ons.gov.uk/ons/rel/wellbeing/measuring-national-well-being/commuting-and-personal-well-being--2014/art-commuting-and-personal-well-being.html

Ogilvie, D, Griffin, S, Jones, A, … Chapman, C (2010) Commuting and health in Cambridge: A study of a “natural experiment” in the provision of new transport infrastructure. *BMC Public Health*, **10**. doi:10.1186/1471-2458-10-703.

Ohta, M, Mizoue, T, Mishima, N, and Ikeda, M (2007) Effect of the physical activities in leisure time and commuting to work on mental health. *Journal of Occupational Health*, **49**(1), 46–52. doi:10.1539/joh.49.46.

Olsson, LE, Gärling, T, Ettema, D, Friman, M, and Fujii, S (2013) Happiness and Satisfaction with Work Commute. *Social Indicators Research*, **111**(1), 255–263. doi:10.1007/s11205-012-0003-2.

Page, NC, and Nilsson, VO (2017) Active commuting: Workplace health promotion for improved employee well-being and organizational behavior. *Frontiers in Psychology*, **7**(JAN). doi:10.3389/fpsyg.2016.01994.

Parloff, MB, Kelman, HC, and Frank, JD (1954) Comfort, effectiveness, and self-awareness as criteria of improvement in psychotherapy. *The American Journal of Psychiatry*, **111**(5), 343–352. doi:10.1176/AJP.111.5.343.

Pearlin, LI, and Schooler, C (1978) The structure of coping. *Journal of Health and Social Behavior*, **19**(1), 2–21. doi:10.2307/2136319.

Pucci, G, Reis, RS, Rech, CR, and Hallal, PC (2012) Quality of life and physical activity among adults: Population-based study in Brazilian adults. *Quality of Life Research*, **21**(9), 1537–1543. doi:10.1007/s11136-011-0083-5.

QualityMetrics (2008) *A manual for users of the SF-8_health survey quality*, Metrics.

Radloff, LS (1977) The CES-D Scale: A Self-Report Depression Scale for Research in the General Population. *Applied Psychological Measurement*, **1**(3), 385–401. doi:10.1177/014662167700100306.

Raitasalo, R (1995) *Elämänhallinta sosiaalipolitiikan tavoitteena. (Coping as the target of social policy)*, Helsinki: The Social Insurance Institution, Finland, Studies in Social Securitary and Health 1.

Rasciute, S, and Downward, P (2010) Health or happiness? What is the impact of physical activity on the individual? *Kyklos*, **63**(2), 256–270.

Reich, B, Niederseer, D, Loidl, M, … Niebauer, J (2020) Effects of active commuting on cardiovascular risk factors: GISMO—a randomized controlled feasibility study. *Scandinavian Journal of Medicine and Science in Sports*, **30**(S1), 15–23. doi:10.1111/SMS.13697.

Rissel, C, Greaves, S, Wen, LM, Capon, A, Crane, M, and Standen, C (2013) Evaluating the transport, health and economic impacts of new urban cycling infrastructure in Sydney, Australia - Protocol paper. *BMC Public Health*, **13**(1). doi:10.1186/1471-2458-13-963.

Russell, DW (1996) UCLA Loneliness Scale (Version 3): Reliability, validity, and factor structure. *Journal of Personality Assessment*, **66**(1), 20–40.

Russell, JA, and Lanius, UF (1984) Adaptation level and the affective appraisal of environments. *Journal of Environmental Psychology*, **4**(2), 119–135. doi:10.1016/S0272-4944(84)80029-8.

Ryckman, RM, Robbins, MA, Thornton, B, and Cantrell, P (1982) Development and validation of a physical self-efficacy scale. *Journal of Personality and Social Psychology*, **42**(5), 891.

Sattler, MC, Färber, T, Traußnig, K, … van Poppel, MNM (2020) Cross-sectional association between active commuting and perceived commuting stress in Austrian adults: Results from the HOTway study. *Mental Health and Physical Activity*, **19**. doi:10.1016/j.mhpa.2020.100356.

Scarabottolo, CC, Cyrino, ES, Nakamura, PM, … Christofaro, DGD (2019) Relationship of different domains of physical activity practice with health-related quality of life among community-dwelling older people: a cross-sectional study. *BMJ Open*, **9**(6), e027751. doi:10.1136/bmjopen-2018-027751.

Scarabottolo, CC, Tebar, WR, Gobbo, LA, … Christofaro, DGD (2022) Analysis of different domains of physical activity with health-related quality of life in adults: 2-year cohort. *Health and Quality of Life Outcomes*, **20**, 1–9. doi:10.1186/s12955-022-01981-3.

Schwanen, T, and Mokhtarian, PL (2004) The extent and determinants of dissonance between actual and preferred residential neighborhood type. *Environment and Planning B: Planning and Design*, **31**(5), 759–784. doi:10.1068/B3039.

Sener, IN, and Lee, RJ (2017) Active travel behavior in a border region of Texas and New Mexico: Motivators, deterrents, and characteristics. *Journal of Physical Activity and Health*, **14**(8), 636–645. doi:10.1123/jpah.2016-0503.

Sener, IN, Lee, RJ, Simek, C, Sánchez, A, and Aldrete, RM (2017) Examining multimodal transportation in the el paso, texas, region: Regional transportation survey results. *Transportation Research Record*, **2654**(1), 55–64. doi:10.3141/2654-07.

Shacham, S (1983) A shortened version of the Profile Mood States. *Journal of Personality Aassessment*.

Singleton, PA (2019) Walking (and cycling) to well-being: Modal and other determinants of subjective well-being during the commute. *Travel Behaviour and Society*, **16**, 249–261. doi:10.1016/j.tbs.2018.02.005.

Smith, JM (2012) Toward a better understanding of loneliness in community-dwelling older adults. *Journal of Psychology: Interdisciplinary and Applied*, **146**(3), 293–311. doi:10.1080/00223980.2011.602132.

Smith, O (2017) Commute well-being differences by mode: Evidence from Portland, Oregon, USA. *Journal of Transport and Health*, **4**, 246–254. doi:10.1016/j.jth.2016.08.005.

Solem, PE (2003) *Forskningsinstrumentene i NorLAG [The Research Instruments in the NorLAGstudy].*, Oslo: Norwegian Social Research.

St-Louis, E, Manaugh, K, Van Lierop, D, Student, P, and El-Geneidy, A (2014) The happy commuter: A comparison of commuter satisfaction across modes. *Transportation Research Part F: Traffic Psychology and Behaviour*, **26**, 160–170.

Sullivan, M, Karlsson, J, and Ware, JE (1995) The Swedish SF-36 Health Survey-I. Evaluation of data quality, scaling assumptions, reliability and construct validity across general populations in Sweden. *Social Science and Medicine*, **41**(10), 1349–1358. doi:10.1016/0277-9536(95)00125-Q.

Taylor, M, Brice, J, Buck, N, and Prentice-Lane, E (2001) *British Household Panel Survey-User-Manual-Volume A: Introduction, Technical Report and Appendices*, Colchester: Institute for Social and Economic Research, University of Essex.

Tennant, R, Hiller, L, Fishwick, R, … Stewart-Brown, S (2007) The Warwick-Dinburgh mental well-being scale (WEMWBS): Development and UK validation. *Health and Quality of Life Outcomes*, **5**. doi:10.1186/1477-7525-5-63.

The EuroQoL Group (1990) EuroQol - a new facility for the measurement of health-related quality of life. *Health Policy*, **16**(3), 199–208. doi:10.1016/0168-8510(90)90421-9.

The Whoqol Group (1998) The World Health Organization quality of life assessment (WHOQOL): Development and general psychometric properties. *Social Science & Medicine*, **46**(12), 1569–1585.

Thompson, ER (2007) Development and validation of an internationally reliable short-form of the Positive and Negative Affect Schedule (PANAS). *Journal of Cross-Cultural Psychology*, **38**(2), 227–242. doi:10.1177/0022022106297301.

Trost, SG, Owen, N, Bauman, AE, Sallis, JF, and Brown, W (2002) Correlates of adults’ participation in physical activity: Review and update. *Medicine & Science in Sports & Exercise*, **34**(12), 1996–2001.

Vancampfort, D, Smith, L, Stubbs, B, … Koyanagi, A (2018) Associations between active travel and physical multi-morbidity in six low- and middle-income countries among community-dwelling older adults: A cross-sectional study. *PLoS ONE*, **13**(8). doi:10.1371/journal.pone.0203277.

Västfjäll, D, Friman, M, Gärling, T, and Kleiner, M (2002) The measurement of core affect: a Swedish self-report measure derived from the affect circumplex. *Scandinavian Journal of Psychology*, **43**(1), 19–31. doi:10.1111/1467-9450.00265.

Vuillemin, A, Boini, S, Bertrais, S, … Briançon, S (2005) Leisure time physical activity and health-related quality of life. *Preventive Medicine*, **41**(2), 562–569. doi:10.1016/J.YPMED.2005.01.006.

Wang, X, Yin, C, and Shao, C (2021) Relationships among haze pollution, commuting behavior and life satisfaction: A quasi-longitudinal analysis. *Transportation Research Part D: Transport and Environment*, **92**. doi:10.1016/j.trd.2021.102723.

Ware, J, Kosinski, M, Dewey, J, … Dewey, J (2001) How to score and interpret single-item health status measures: a manual for users of the SF-8^TM^ Health Survey.

Ware, JE, and Gandek, B (1998) Overview of the SF-36 Health Survey and the International Quality of Life Assessment (IQOLA) Project. *J Clin Epidemiol*, **51**(11), 903–912.

Ware, JE, Kosinski, M, and Keller, SD (1996) A 12-Item Short-Form Health Survey: Construction of Scales and Preliminary Tests of Reliability and Validity., **34**(3), 220–233.

Ware, JE, Snow, KK, Kosinski, M, and Gandek, B (1993) *SF-36 health survey. Manual and Interpretation guide, 2.*

Ware, JEJr, Kosinski, M, Turner-Bowker, DM, and Gandek, B (2002) User’s Manual for the SF-12v2® Health Survey With a Supplement Documenting SF-12® Health Survey., Lincoln, RI: QualityMetric Incorporated.

Watson, D, Clark, LA, and Tellegen, A (1988) Development and Validation of Brief Measures of Positive and Negative Affect: The PANAS Scales. *Journal of Personality and Social Psychology*, **54**(6), 1063–1070. doi:10.1037/0022-3514.54.6.1063.

West, LA, Cole, S, Goodkind, D, and He, W (2014) *65+ in the United States: 2010 (Current Population Reports)*, Washington, DC: U.S.: Census Bureau.

Yang, Y, Langellier, BA, Stankov, I, Purtle, J, Nelson, KL, and Diez Roux, AV (2019) Examining the possible impact of daily transport on depression among older adults using an agent-based model. *Aging & Mental Health*, **23**(6), 743–751. doi:10.1080/13607863.2018.1450832.
